# Tendinopathy: Types, Epidemiology, Molecular Mechanisms, and Future Precision‐Oriented Therapeutic Strategies

**DOI:** 10.1002/mco2.71098

**Published:** 2026-10-29

**Authors:** Yang Wu, Junyi Long, Meng Yang, Hongjie Huang, Jianquan Wang

**Affiliations:** ^1^ Department of Sports Medicine Beijing Key Laboratory of Sports Injuries Peking University Third Hospital Beijing China; ^2^ Institute of Sports Medicine of Peking University Beijing China; ^3^ Engineering Research Center of Sports Trauma Treatment Technology and Devices in Ministry of Education Beijing China; ^4^ Beijing University of Chinese Medicine Affiliated Dongzhimen Hospital Beijing China

**Keywords:** tendinopathy, pathophysiology, mechanical stress, inflammation, angiogenesis, translational therapy

## Abstract

Tendinopathy represents a heterogeneous spectrum of tendon disorders characterized by persistent pain, impaired function, and progressive disruption of tissue homeostasis. Rather than a single degenerative entity, tendinopathy reflects the interaction between mechanical adaptation failure, immune dysregulation, extracellular matrix remodeling, and systemic metabolic influences. However, its pathophysiological mechanisms remain incompletely understood. Tendinopathy can affect multiple anatomical sites, such as the rotator cuff, Achilles tendon, and patellar tendon, and has a high prevalence in both the general population and athletes. Despite decades of research, there is currently a lack of targeted therapies capable of effectively reversing the pathological process, and clinical management is primarily limited to empirical symptomatic treatment. This review systematically synthesizes the epidemiology and risk factors of tendinopathy and proposes a “multidimensional tendinopathy remodeling network” model encompassing mechanotransduction, inflammatory cascades, pathological angiogenesis, and matrix turnover. Furthermore, we discuss emerging approaches for molecular stratification through multimodal imaging and biomarkers, and evaluate precision‐oriented therapeutic strategies, including mechanomodulation, immunomodulation, regenerative medicine, and nano delivery systems. This framework provides a conceptual foundation for understanding the heterogeneity of tendinopathies and facilitates a shift from symptom‐oriented treatment to mechanism‐oriented approaches, potentially leading to precision treatment strategies. At the same time, it highlights the challenges in current translational research and points to future research directions for personalized tendon repair strategies.

## Graphical abstract

This review presents tendinopathy as a heterogeneous disorder driven by dynamic interactions among mechanical sensing, inflammation, angiogenesis and innervation, and extracellular matrix remodeling. Integrating clinical phenotypes with imaging, biomarkers, and multi‐omics may enable molecular subtyping and mechanism‐guided interventions, advancing precision care to improve tendon function and reduce pain and recurrence.

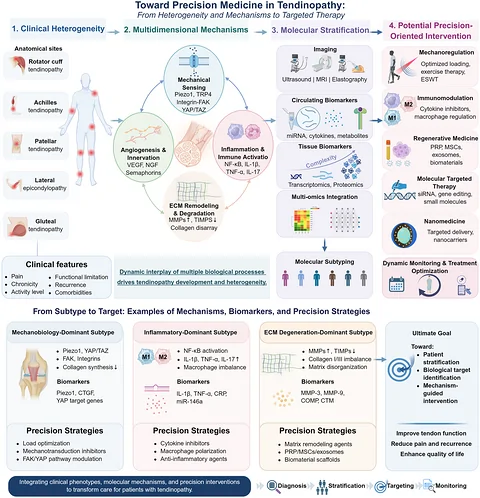

## 1 Introduction

Tendinopathy refers to a complex and multifaceted pathological condition of tendons characterized by pain, functional decline, and reduced exercise tolerance, commonly occurring in the Achilles tendon, rotator cuff, patellar tendon, medial and lateral epicondyles of the elbow, and gluteal tendons [1, 2]. The reported lifetime prevalence of clinically recognized tendinopathy is approximately 6%, although estimates vary considerably according to anatomical site, population characteristics, and diagnostic criteria. Among athletes, the prevalence reaches as high as 50%, primarily involving Achilles tendinopathy in runners, patellar tendinopathy in jumping sports (basketball, volleyball), and rotator cuff and elbow tendinopathy in throwing sports (baseball, tennis). The affected sites vary significantly across different sports [3]. In adults, the high incidence of tendinopathy correlates with multiple factors, including age, gender, obesity, diabetes, and occupation, all of which may increase risk [4, 5, 6, 7]. This condition causes long‐term impairment of physical function, significantly affecting the mental health and quality of life of patients and their families [8]. Beyond the direct medical costs associated with tendinopathy, the disease leads to substantial work absenteeism and reduced productivity, thereby increasing the socioeconomic burden [9].

Currently, the clinical management of tendinopathy remains primarily based on empirical symptomatic treatment, including conventional approaches such as activity modification, physical therapy, oral nonsteroidal anti‐inflammatory drugs (NSAIDs), and local corticosteroid injections. In recent years, regenerative and physical interventions such as platelet‐rich plasma (PRP) injections and extracorporeal shock wave therapy (ESWT) have gradually been incorporated into clinical practice, and novel treatment approaches—represented by stem cells, biomaterials, and gene editing—have also shown promising results in research [10]. Additionally, personalized training protocols, such as self‐regulated training, are recognized as potentially enhancing treatment outcomes for athletes with tendinopathy [11]. However, despite the growing number of treatment options, the efficacy of tendon disorder treatment remains far from satisfactory. Particularly for athletes, the high rates of recurrence and failure to return to sport highlight the limitations of current treatment strategies [12].

The root cause of these challenges lies in our incomplete understanding of the pathophysiological mechanisms underlying tendinopathy. For a long time, both basic research and clinical trials have focused on single factors (whether emphasizing mechanical overload, inflammatory responses, or vascular proliferation), lacking a unified theoretical framework capable of integrating the interactions among multiple factors. Significant heterogeneity exists among different subtypes, such as inflammation‐dominant and degeneration‐dominant types, in terms of molecular characteristics, clinical manifestations, and treatment responses; however, existing diagnostic methods struggle to achieve precise subtyping at an early stage, and there is a lack of clinically validated biomarkers.

Importantly, emerging evidence suggests that tendinopathy should not be considered a single pathological entity but rather a spectrum of biologically distinct conditions. Although patients may present with similar clinical symptoms, the dominant molecular mechanisms driving disease progression may differ substantially; anatomical classification alone is insufficient for guiding individualized therapy. To address these issues, in this review, we first summarize the epidemiological characteristics and risk factors associated with tendinopathy to provide an overview of disease distribution and clinical heterogeneity. We then discuss the multidimensional pathological mechanisms underlying tendinopathy, including mechanical sensing abnormalities, immune dysregulation, vascular–neural remodeling, and extracellular matrix disruption. Subsequently, we summarize current diagnostic strategies, including clinical assessment, multimodal imaging, and emerging biomarkers, and discuss their potential roles in disease stratification. Finally, we review conventional and emerging therapeutic approaches, with particular emphasis on mechanism‐oriented and precision medicine strategies, while highlighting current translational challenges and future research directions (Figure 1).

**FIGURE 1:**
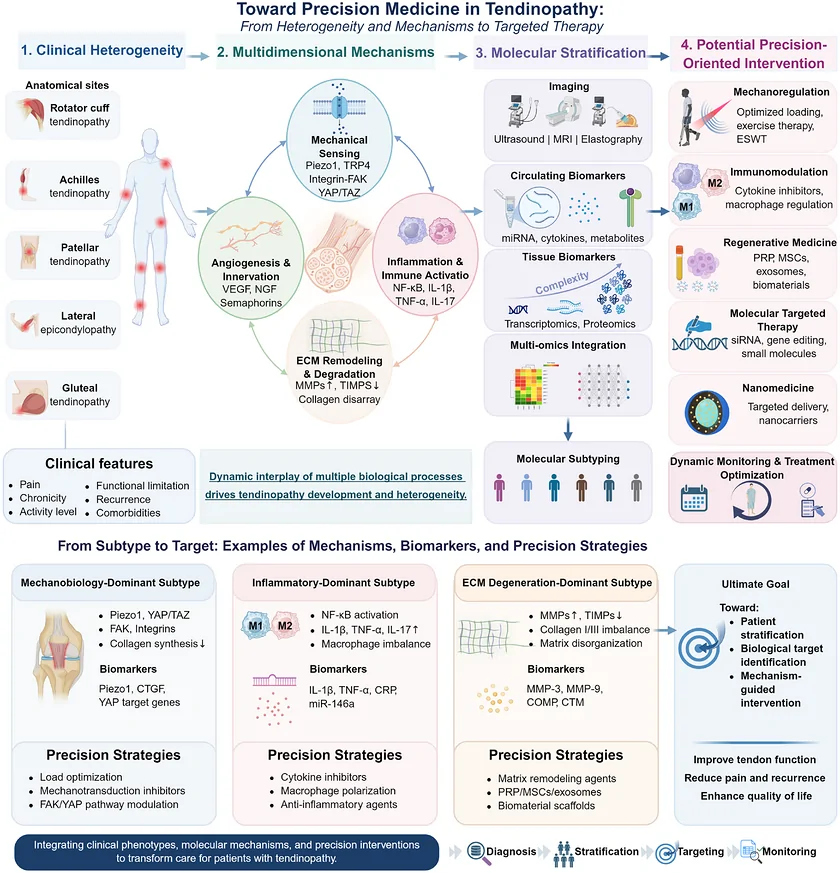
Toward precision‐oriented strategies for heterogeneous tendinopathy management. This figure illustrates the relationship between clinical heterogeneity, multidimensional pathological mechanisms, molecular stratification, and potential mechanism‐oriented interventions. Current approaches and future directions are summarized within a conceptual framework.

## 2 Epidemiology and Risk Factors

Since tendinopathy is widespread in the general population and difficult to cure completely, understanding which groups are at risk and the specific risk factors is essential and serves as a key strategy for preventing and treating this condition.

### 2.1 Demographic Distribution Characteristics

As a common musculoskeletal disorder, the incidence of tendinopathy exhibits significant variation across different age and gender groups. Among adolescents (<18 years old), the prevalence of tendinopathy ranges from 8% to 33%, with a higher proportion observed in males. This may be attributed to their greater susceptibility to structural changes in tendons during sports activities and the rapid growth associated with adolescence [13]. Adolescent males are more susceptible to overuse and mechanical stress during sports activities, further elevating their risk of tendinopathy [6]. While the incidence of tendinopathy remains high among adults (18–65 years), females outnumber males in this age group. In adults, the high prevalence of tendinopathy correlates with multiple factors, including but not limited to age, gender, obesity, and diabetes [14]. Women, in particular, may be more susceptible to tendinopathy due to hormonal fluctuations [4]. Their unique physiological structure and movement patterns make them more prone to tendon injuries during exercise or physical activities [5]. Future research should further explore the specific roles of these factors in the pathogenesis of tendinopathy to develop more effective prevention and treatment strategies.

A recent 25‐year population‐based study using electronic medical records from 36,970 patients across 47 Hong Kong public hospitals (2000–2024) further demonstrated marked epidemiological heterogeneity by anatomical site. The study reported overall, age‐standardized, and sex‐standardized annual incidence rates for 10 tendinopathies and seven nontraumatic tendon ruptures. The mean annual incidence was 15.48 ± 6.33 cases per 100,000 persons for tendinopathies and 5.57 ± 0.93 cases per 100,000 persons for nontraumatic tendon ruptures. Lateral epicondylitis (8.84 ± 3.31 per 100,000) and Achilles tendinopathy (3.09 ± 1.59 per 100,000) were the predominant tendinopathies, whereas rotator cuff rupture (1.90 ± 0.87 per 100,000) and Achilles tendon rupture (1.73 ± 0.37 per 100,000) were the most frequent nontraumatic ruptures. Age‐ and sex‐specific analyses further showed that most tendinopathies were more frequent among adults aged 40–59 years, except patellar and Achilles tendinopathies, which predominated in younger groups; several tendinopathies were more common in females, whereas patellar tendinopathy and multiple tendon ruptures were more common in males [15].

Moreover, tendinopathy is closely linked to occupational factors, particularly in high‐load or repetitive‐motion occupations. For instance, work activities involving heavy lifting and the use of vibrating tools are associated with medial epicondylitis [16]. These findings underscore that repetitive motions or specific physical factors in the workplace are significant contributors to tendinopathy. Individuals whose occupations involve physical activity exhibit higher rates of tendinopathy. Tennis players, due to the nature of their sport, are prone to rotator cuff injuries, often resulting from overuse and internal shoulder impingement, a condition particularly prevalent among younger athletes [17]. Achilles tendinopathy in runners is also attributed to the high‐intensity loads inherent in their sport. These athletes often experience intermittent episodes of tendinopathy, indicating that a history of previous tendon injuries is also a significant risk factor for tendinopathy [18]. These findings not only enhance our understanding of the etiology of tendinopathy but also provide important reference points for prevention and treatment. Future research should continue to explore the specific mechanisms underlying these factors to enable more effective intervention strategies.

### 2.2 Risk Factor Classification and Mechanisms

The onset of tendinopathy is the combined result of the interaction between internal factors (abnormal joint anatomy and function, metabolic disorders, and genetic factors) and external factors (inappropriate mechanical loading, medication use, poor work posture, and smoking). These factors do not exist in isolation but rather overlap and reinforce one another, ultimately exceeding the tendon's self‐repair threshold, leading to the onset and progression of the disease. Furthermore, the incidence varies across different anatomical sites (Figure 2).

**FIGURE 2:**
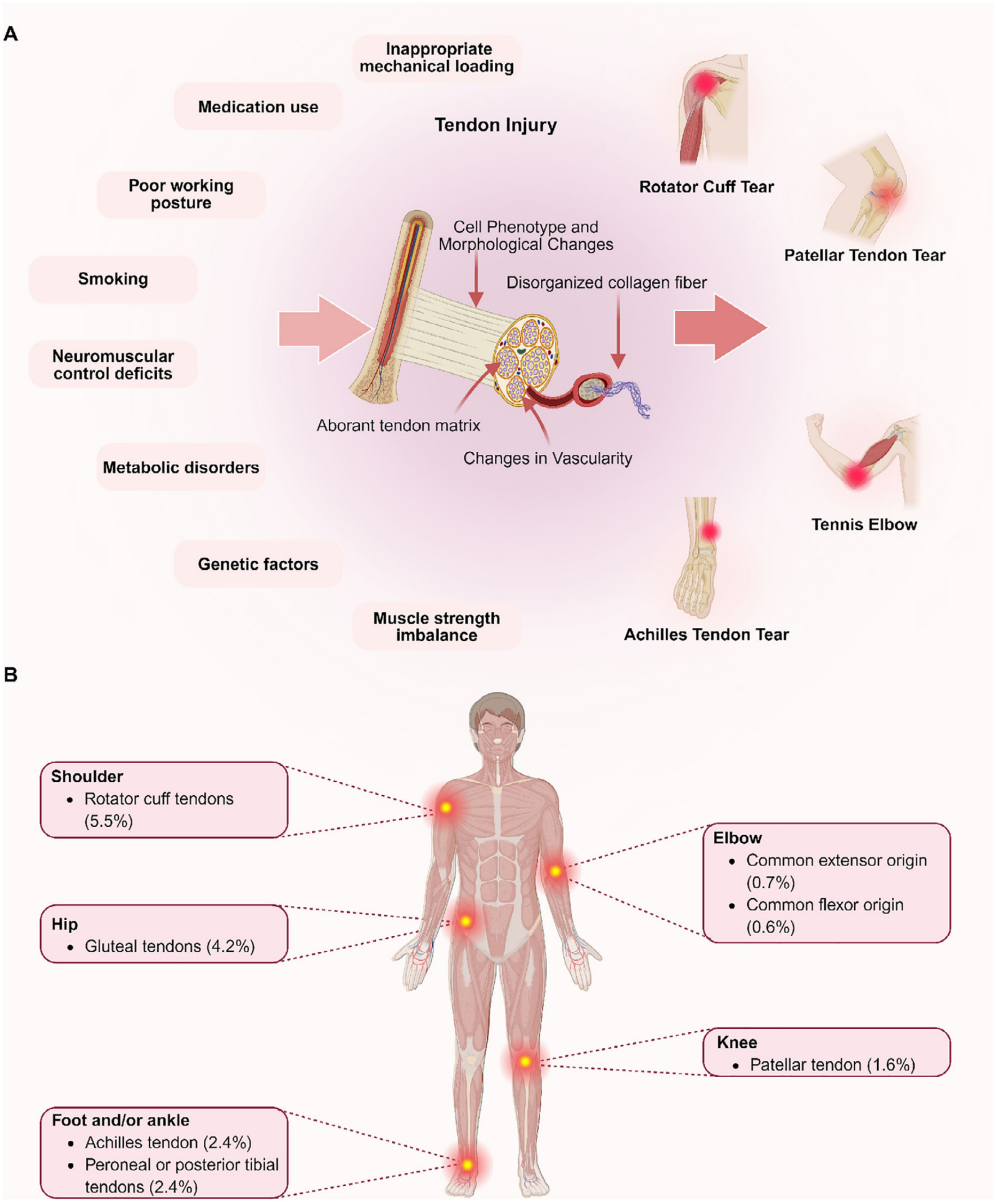
Risk factors and anatomical distribution associated with tendinopathy. (A) The pathogenesis of tendinopathy is the result of interactions between intrinsic factors and extrinsic factors. (B) The incidence of tendinopathy varies across different anatomical sites.

#### 2.2.1 Intrinsic Risk Factors

Although tendinopathy predominantly occurs at various joints, its risk factors extend beyond the joint itself and are closely linked to other bodily systems. This review summarizes the following intrinsic risk factors: joint anatomical and functional abnormalities, metabolic disorders, and genetic factors.

The foremost intrinsic risk factor for tendinopathy is joint anatomical and functional abnormalities, encompassing restricted joint mobility, muscle strength imbalances, and neuromuscular control deficits. Restricted joint mobility stands as a significant risk factor for tendinopathy. Specifically, diminished shoulder joint mobility correlates closely with sports injuries. For instance, among baseball players, restricted internal rotation of the shoulder joint is associated with an increased risk of throwing shoulder injuries [19]. Muscle strength imbalances represent another significant risk factor for tendinopathy. Muscular strength disparities may contribute to joint instability, thereby elevating injury susceptibility [20]. Deficits in neuromuscular control similarly constitute a critical risk factor for tendinopathy. Research indicates that impaired neuromuscular control may diminish joint stability. For instance, in the shoulder joint, muscle preactivation and reactive recruitment are important in maintaining stability, while posterior shoulder tightness can cause scapular malposition, further affecting shoulder function and stability [21]. Thus, joint function and stability are influenced by multiple factors, ultimately elevating the risk of tendinopathy.

Intrinsic risk factors for tendinopathy also include metabolic disorders such as diabetes, hyperlipidemia, and chronic kidney disease, which elevate susceptibility by impairing tendon repair capacity. Fluctuating blood glucose levels in diabetic patients significantly impair tendon healing [22]. Obesity and elevated cholesterol levels are also recognized risk factors for tendinopathy, as they not only reduce tendon blood supply and increase metabolic burden [23] but also exert pro‐inflammatory and vascular endothelial toxic effects, thereby heightening the risk of tendon pathology [24, 25]. The accumulation of metabolic waste products may also impair tendon repair capacity. Studies indicate a significantly elevated risk of tendinopathy in patients with diabetes complicated by chronic kidney disease [26]. As can be seen, diabetes is more strongly associated with Achilles tendinopathy than with rotator cuff tendinopathy, likely due to the relatively hypovascular nature of the Achilles tendon, which exacerbates the accumulation of advanced glycation end‐products (AGEs) and impairs collagen cross‑linking. In contrast, hyperlipidemia shows a stronger correlation with rotator cuff tears, possibly because lipid infiltration and inflammatory cytokines in the subacromial space create a pro‑catabolic microenvironment specific to the supraspinatus tendon. Thus, early identification and intervention targeting these risk factors could help reduce the incidence of tendinopathy and improve patients' quality of life [27].

Among the intrinsic risk factors for tendinopathy, genetic factors are also crucial, particularly mutations in the collagen type V alpha 1 chain (COL5A1), the metalloproteinases (MMPs) family, the tissue inhibitors of metalloproteinases (TIMPs) family, and Tenascin‐C (TNC). The V‐type collagen encoded by the COL5A1 gene serves as a key regulator of collagen fiber assembly and diameter. Its variants may alter tendon structure, thereby increasing the risk of tendon injury [28]. MMP3, a key member of the matrix metalloproteinase family, participates in maintaining extracellular matrix integrity; its variants may affect tendon repair capacity [29]. Certain MMP3 gene variants interact with the rs12722 variant of the COL5A1 gene, and this interaction may increase the risk of Achilles tendinopathy [30]. Additionally, studies have found that the rs4789932 variant of the TIMP2 gene is closely associated with the risk of chronic Achilles tendinopathy [31]. Moreover, TIMP2, as a metalloproteinase inhibitor, maintains extracellular matrix homeostasis by suppressing metalloproteinase (MMP) function, thereby influencing tendon health [32]. Polymorphisms in the TNC gene are also associated with the risk of failed tendon healing, suggesting the TNC gene may act as a critical mediator in tendon repair processes [33]. On the other hand, epigenetics has also demonstrated significance in tendinopathy research. Studies indicate that alterations in DNA methylation patterns may increase tendinopathy risk [34]. MicroRNAs (miRNAs) play a crucial role in the pathogenesis of osteoarthritis (OA) by regulating gene expression and extracellular matrix remodeling [35]. Notably, interactions between genes and miRNAs are also recognized to influence tendon function [36]. Collectively, genetic polymorphisms or interactions constitute the essential contributors to the onset and progression of tendinopathy. In‐depth research in this area not only aids in elucidating the genetic mechanisms underlying tendinopathy but may also provide novel insights for personalized prevention and treatment strategies.

#### 2.2.2 External Risk Factors

Beyond the various intrinsic factors that cause or trigger tendinopathy, external risks also warrant significant attention. Here, we summarize the following key external risk factors, primarily including improper mechanical loading, medication use, poor work posture, and smoking.

Among external risk factors for tendinopathy, improper mechanical loading is a significant aspect, manifesting as repetitive microdamage caused by overuse, sudden increases in exercise intensity, incorrect exercise technique, and insufficient recovery. Athletes, in particular, are prone to lower‐limb overuse injuries when performing repetitive landing movements [37]. Adolescent athletes participating in throwing sports are prone to forearm flexor tendon injuries due to repetitive motions and inadequate rest, further illustrating the impact of overuse and insufficient recovery on tendon health [38]. Additionally, degenerative changes in tendons and failed healing responses are closely linked to insufficient recovery, collectively contributing to the onset and progression of tendinopathy [39]. Second, incorrect athletic technique is another significant factor contributing to tendinopathy [40, 41]. It is evident that external risk factors for tendinopathy encompass various aspects of improper mechanical loading, which have been extensively confirmed and discussed across different studies.

Moreover, the occurrence of tendinopathy is closely associated with medication use. Among these, the use of fluoroquinolone antibiotics and glucocorticoids is considered one of the primary factors increasing the risk of tendon injury. People exposed to fluoroquinolone antibiotics have an increased risk of Achilles tendon rupture, particularly within the first month of treatment (with a risk ratio ranging from 1.1 to 7.1) [42]. Fluoroquinolone drugs, such as levofloxacin and ofloxacin, are associated with a higher risk of tendon injury, a risk that increases with higher drug doses and longer duration of use [43]. Thus, we propose that it is necessary to provide tendinopathy management education for patients taking these medications or to seek alternative therapies.

Poor work postures and high‐intensity physical labor are also recognized as potential risk factors for tendon injuries. Occupational tendinopathy is more prevalent among workers engaged in physically demanding jobs, such as firefighters and military personnel, where prolonged periods of strenuous physical activity heighten the risk of tendon damage [44]. Musicians are susceptible to musculoskeletal disorders, particularly upper limb tendinopathy and compression syndromes, due to prolonged repetitive motions and poor posture [45]. Thus, improving work environments and posture to reduce unnecessary strain on muscles and tendons constitutes a crucial preventive measure against tendon injuries.

Surprisingly, smoking also represents a significant risk factor for tendon injuries. It is closely associated with the development of various tendon disorders, including rotator cuff tears and other tendinopathies. Studies indicate that smokers experience poorer recovery outcomes and higher pain levels following shoulder surgery, further underscoring smoking's detrimental impact on tendon health [46].

## 3 Pathophysiological Mechanisms

Tendinopathy is increasingly recognized as a heterogeneous and dynamic disorder rather than a simple consequence of mechanical overload or tissue degeneration [47, 48, 49]. This section summarizes the multidimensional biological mechanisms underlying tendinopathy progression, focusing on the interactions among mechanical‐sensing abnormalities, inflammatory responses, vascular‐neural remodeling, extracellular matrix (ECM) disruption, and metabolic adaptation. We first describe alterations in tendon structure and homeostasis, followed by an integrated discussion of the remodeling network that connects these biological processes. Understanding these interconnected mechanisms provides a theoretical basis for molecular stratification and the development of mechanism‐oriented therapeutic strategies.

### 3.1 Imbalance in Tendon Structure and Homeostasis

The “multidimensional tendinopathy remodeling network” is rooted in an imbalance in tendon structure and homeostasis. It is crucial to understand the process by which a healthy tendon's structure and function change into a pathological state [3] (Figure 3).

**FIGURE 3:**
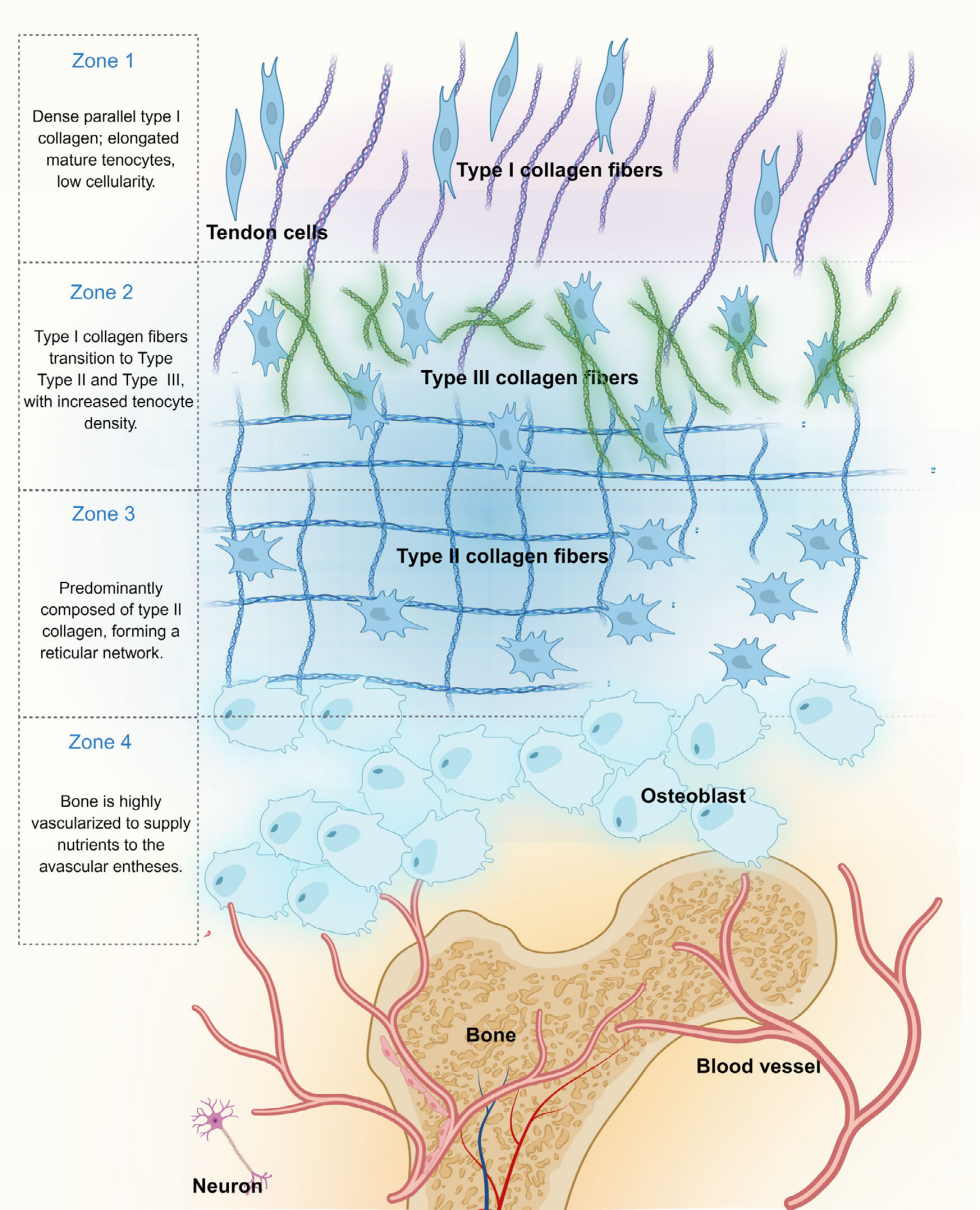
The strata and a collagen gradient of fibrocartilaginous enthesis. The four consecutive gradient layers where the tendon transitions from “pure tendon tissue” to “bone tissue” at its attachment to the bone surface; if this layered structure is disrupted, it constitutes the anatomical and histological basis for the onset and progression of tendon disorders.

Normal tendons comprise highly ordered fiber networks primarily composed of fibrillar collagen. Tendon cells align along collagen fibers, maintaining equilibrium between extracellular matrix (ECM) synthesis and degradation. Within the cellular and extracellular mechanisms of tendons, fibrillar collagen serves as a core component in establishing tendon structure and function by regulating fiber formation and intercellular communication. Deficiency in fibrillar collagen leads to alterations in tendon cell morphology and tissue organization, subsequently affecting intercellular communication and the hierarchical structure of the tissue [50, 51]. Tendinocytes are the primary cell type in tendon tissue, responsible for maintaining tendon structure and function. Tendinocytes not only provide structural support but also modulate immune surveillance; their interactions with immune cells are essential for tendon inflammatory responses and repair processes [52]. Tendon stem/progenitor cells (TSPCs) also play a particularly vital role in tendon tissue regeneration. Possessing potent differentiation potential, they can differentiate into tendon cells, significantly contributing to the healing process of tendon injuries [53]. They also accelerate tendon healing by secreting exosomes and other bioactive molecules that promote tendon cell proliferation and migration [54, 55]. TSPC‐derived exosomes (TSPC‐Exos) are taken up by fibroblasts and promote cell proliferation and migration in a dose‐dependent manner; this process may depend on the activation of the PI3K/AKT and MAPK/ERK1/2 signaling pathways [54]. The photopolymerizable hyaluronic acid (p‐HA) loaded with TSPC‐Exos (pHA‐TSPC‐Exos) scaffold has been shown to promote tendon repair in a rat model. miR‐144‐3p is enriched in TSPC‐Exos and promotes the proliferation and migration of tendon fibroblasts by targeting AT‐rich interaction domain 1A (ARID1A) [55]. Matrix‐associated proteins are a class of nonstructural secreted proteins within the extracellular matrix. Among these, the cystine‐rich acidic secretory protein (SPARC) is of paramount importance in tendon mechanobiology, particularly in tissue maturation, homeostasis, and insertion site development: SPARC deficiency leads to tendon tissue hypoplasia and impaired maturation, subsequently affecting type I collagen synthesis [56]. Finally, tendon tissue also contains endothelial cells, macrophages, nerve cells, adipocytes, and TSPC subpopulations, which collectively participate in tendon repair and regeneration [57, 58]. Thus, the cellular composition and functions of tendon tissue are complex and diverse, with various cell types playing indispensable roles in structural maintenance, immune surveillance, and regeneration.

Structural alterations in tendons represent a complex pathological process involving multiple aspects such as collagen fiber damage, increased proportion of type III collagen, and glycosaminoglycan accumulation. Research indicates that adaptive changes in tendons under load involve the synthesis and organization of type I collagen [59]. Regarding mechanical signaling, tendon cells respond to mechanical loads through pathways such as integrins, which regulate mRNA translation and protein synthesis, thereby influencing collagen expression and tendon adaptation [60]. Collagen denaturation and degradation are indispensable for tendinopathy progression. Prolonged running induces increased collagen degradation, leading to accumulation of degenerated collagen and tendinopathy progression [61]. Collagen molecules undergo microstructural damage during cyclic fatigue loading, and these alterations may constitute one mechanism underlying tendinopathy development [62, 63]. Moreover, excessive mechanical loading can cause structural damage and functional deterioration in tendons [64]. Additionally, hypoxic environments promote inflammatory cytokine expression and tendon cell apoptosis while increasing type III collagen synthesis through activation of the mitogen‐activated protein kinase (MAPK)‐dependent pathway. These alterations in collagen fiber structure directly reduce tendon mechanical strength, further accelerating lesion progression [65]. Glycosaminoglycan accumulation correlates positively with clinical pain levels, compromising tendon structural integrity and potentially exacerbating disease progression by altering tissue biomechanics. In summary, structural and homeostatic alterations in tendinopathy involve multiple biological processes. These changes not only compromise tendon mechanical properties but also correlate closely with clinical symptoms. Deepening our understanding of the interaction mechanisms between tendon cells and other cell types could provide novel insights and strategies for treating and regenerating tendon injuries [66, 67].

### 3.2 A Multidimensional Remodeling Network Underlying Tendinopathy Progression

Traditional models of tendinopathy progression have largely emphasized a linear transition from mechanical overload to structural degeneration [68]. However, increasing evidence indicates that tendinopathy is not a simple sequential process but rather a dynamic and heterogeneous disease state driven by continuous interactions among mechanical sensing, immune regulation, vascular–neural remodeling, extracellular matrix (ECM) turnover, and metabolic adaptation. Here, we provide a conceptual framework toward mechanism‐informed and precision‐oriented management of tendinopathy (Figure 4) and compare it with several existing models (Table 1). Importantly, the relative contribution of each component is not constant but varies according to anatomical location, biological phenotype, genetic background, metabolic status, and environmental exposure. Hence, we suggest that tendinopathy should be considered not merely as a degenerative consequence of mechanical overload, but as a network disease characterized by context‐dependent biological interactions. This perspective provides a conceptual foundation for molecular stratification and precision treatment strategies by identifying dominant pathological nodes that may serve as therapeutic targets, and we summarized the mechanism of some core pathways in tendinopathy (Table 2). From our perspective, the major limitation of existing models is not the lack of individual mechanisms, but the insufficient integration of these mechanisms into a unified biological context.

**FIGURE 4:**
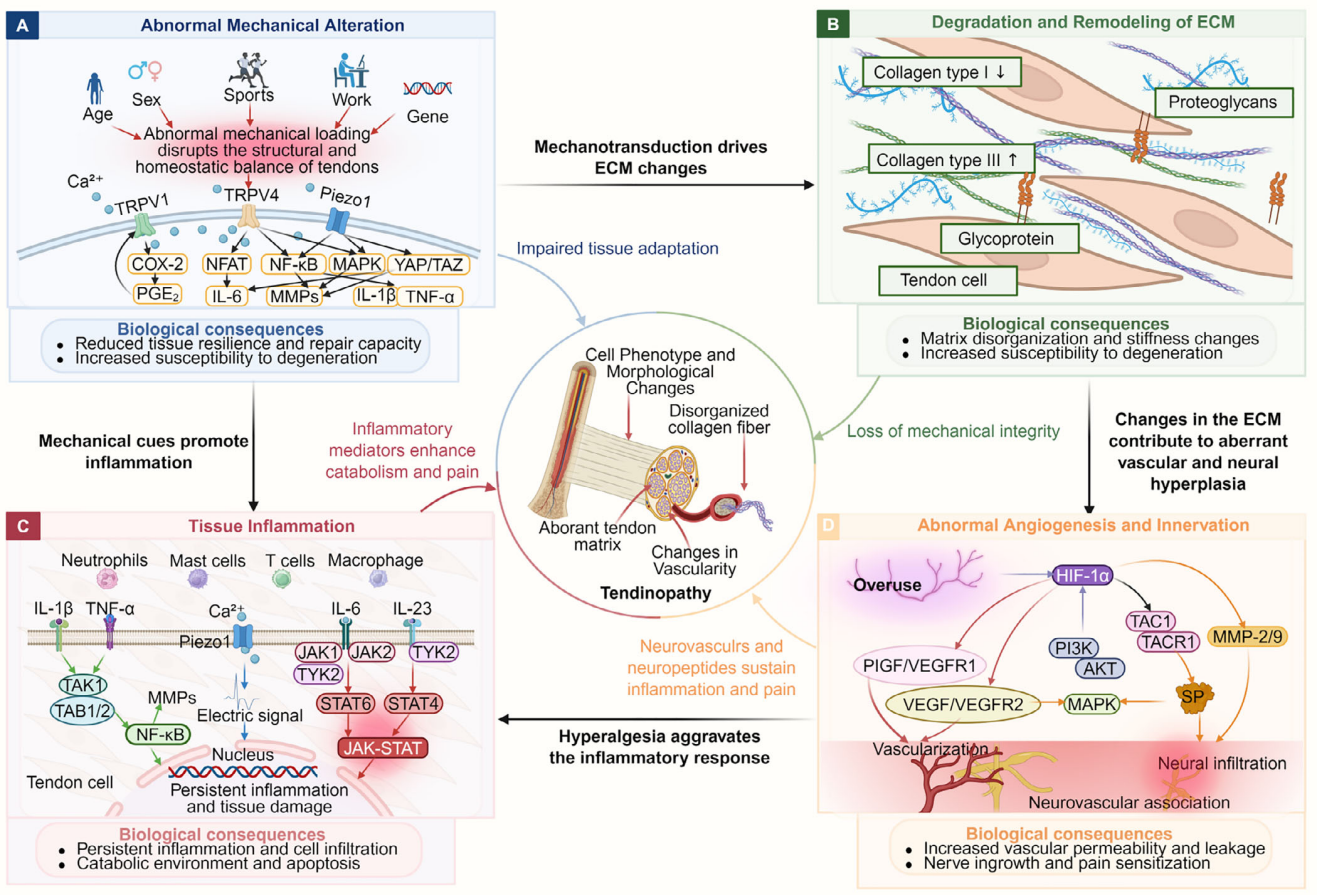
Multidimensional remodeling network underlying tendinopathy progression. Tendinopathy progression is regulated by dynamic interactions among mechanical sensing, inflammatory activation, angiogenesis and innervation, and ECM remodeling.

**TABLE 1:** Comparison of existing models and multidimensional remodeling network.

	Core concept	Limitation	Advancement
Cook–Purdam continuum [68]	Reactive—dysrepair—degeneration	Mainly structural progression	Does not explain molecular heterogeneity
Inflammatory model [97, 147]	Cytokine‐driven pathology	Cannot explain mechanical adaptation	Limited therapeutic stratification
Degenerative model [14, 61, 62, 63, 64, 65]	ECM failure	Static view	Lack dynamic interaction
Multidimensional remodeling network [69, 70, 71, 72, 73, 74, 75, 76, 77, 78, 79, 80, 81, 82, 83, 84, 85, 86, 87, 88, 89, 90, 91, 92, 93, 94, 95, 96, 97, 98, 99, 100, 101, 102, 103, 104, 105, 106, 107, 108, 109, 110, 111, 233]	Dynamic interaction among mechanical, immune, ECM, vascular‐neural modules	Requires more validation	May facilitate future molecular stratification and precision‐oriented therapy development

Data source: Published literature cited in the table and the authors' synthesis; no clinical registry data were used. The “multidimensional remodeling network” is a conceptual framework proposed in this review.

**TABLE 2:** Major signaling pathways, core mechanisms, and pathological consequences in tendinopathy.

Pathway/Target		Core mechanism in tendinopathy	Major pathological involvement
Mechanical signal transduction	Piezo1/TRPV4 [69, 73, 74, 86, 249, 250]	Transduces mechanical overload via Ca^2^^+^ influx	Overload sensing, inflammation initiation
	Integrin‑FAK/YAP‑TAZ [70, 71, 72]	Converts mechanical signals into cellular responses	Mechanotransduction, fibrosis
Inflammatory response	NF‐κB/MAPK/JAK‐STAT [76, 89, 94‑99]	Initiates and amplifies pro‑inflammatory cytokine cascades	Inflammation, ECM breakdown, immune infiltration
	IL‐17/IL‐17A [90, 91, 235, 244]	Drives chronic synovial‑like inflammation, promotes ectopic ossification	Chronic inflammation, heterotopic ossification, fibrosis
	IL‐33/IL‐1β/IL‐6 [88, 147, 155, 245]	Induces tenocyte apoptosis and catabolic shift	Degenerative remodeling, acute/chronic pain
	TNF‐α [92]	Amplifies local inflammation via MMP upregulation and synergistic crosstalk with IL‑17, accelerating matrix breakdown	Inflammation amplification, matrix degradation, apoptosis
	Chemokine axis (CCL2/CXCL1) [76, 86]	Chemoattracts monocytes/macrophages and neutrophils to the lesion, establishing a positive feedback loop that sustains inflammation	Immune cell chemotaxis, persistent inflammation
Angiogenesis and innervation	VEGF [100, 102, 246‑248, 252]	Stabilized under hypoxia to drive pathological angiogenesis and sensory nerve ingrowth; also modulates metabolic adaptation and ossification	Abnormal angiogenesis and innervation, ectopic ossification
	SP/NGF/CGRP [104‑106]	Sensitizes nociceptive afferents and promotes neurogenic inflammation; couples with ECM remodeling via NGF/CGRP signaling.	Neural ingrowth, pain sensitization, neurogenic inflammation
Remodeling of ECM	MMPs/TIMPs [29‑32, 77‑79, 110, 148, 227]	Overactivity of MMP‑2/3/9/13 disrupts collagen networks and cross‑linking	ECM collagen disruption, crosslinking defects
	COL5A1/TNC/MMP3 [28, 30, 33, 227]	Genetic variants alter collagen fibril assembly, ECM turnover, and repair efficiency	Genetic predisposition, structural integrity, repair capacity
	TGF‐β [228, 229]	Stimulates ECM protein synthesis and cellular proliferation, but carries a risk of excessive fibrosis if unregulated	Repair/regeneration, potential fibrotic risk
Metabolic reprogramming	AMPK/mTOR [245, 246]	AMPK activation suppresses mTOR, reducing cell proliferation and inflammatory cytokine release under metabolic stress	Metabolic dysregulation, low‑grade inflammation
Oxidative stress	ROS/oxidative stress pathways [75, 76, 225]	Mitochondria‑derived ROS activate NF‑κB and induce oxidative injury, leading to tenocyte dysfunction and apoptosis.	Oxidative damage, mitochondrial dysfunction, inflammatory amplification
Posttranscriptional regulation	miRNAs (miR‐29a/miR‐144‐3p, et al.) [36, 55, 152, 155]	miR‑29a downregulation favors type III collagen; miR‑144‑3p/148a‑3p modulate cell function and angiogenesis via exosomal transfer	Gene expression control, ECM imbalance, angiogenesis

#### 3.2.1 Pathological Effects of Overloading

Tendon load adaptation is a complex process involving biomechanical and biochemical responses under varying load conditions. Moderate mechanical loading enhances tendon function by promoting collagen turnover and tendon hypertrophy. However, excessive or insufficient loading may induce pathological alterations, subsequently triggering tendinopathy [69]. This pathological effect of overload directly impacts mechanosensitive ion channels such as pressure‐sensitive ion channel protein 1 (PIEZO1), which in turn regulate tendon stiffness [70].

The mechanical signaling pathways in tendinopathy mechanisms constitute a complex biological process involving multiple mechanosensitive ion channels and signaling pathways. The integrin‐focal adhesion kinase (FAK) signaling pathway has a critical bearing on tendinopathy mechanisms. This pathway influences cellular force perception and response by regulating cell adhesion and migration. YAP/TAZ, acting as co‐transcription factors for mechanical signals, contributes to cell proliferation, differentiation, and apoptosis. Separately, YAP/TAZ‐dependent mechanotransduction supports extracellular matrix homeostasis; loss of mechanical tension and reduced YAP/TAZ activity can promote catabolic responses in tendon models [71]. In tendinopathy, abnormal activation of the YAP/TAZ pathway may lead to abnormal cell proliferation and fibrosis, thereby exacerbating the condition [72]. In the musculoskeletal system, PIEZO1 helps maintain muscle and bone density and detects the degree of tendon stretching [73]. PIEZO1 channels interact with the integrin‐FAK signaling pathway to regulate the mechanical properties of the extracellular matrix and are implicated in the progression of tissue fibrosis [74].

Mitochondrial dysfunction has been implicated in tendinopathy [75]. In experimental tendon constructs, mechanical overload disrupts mitochondrial networks and induces the release of extracellular mitochondrial particles that promote macrophage chemotaxis and inflammatory responses [76]. Independent evidence implicates IKKβ/NF‐κB signaling in chronic tendon disease [77]. Human Achilles tendon studies demonstrate distinct metalloproteinase and inhibitor expression profiles in painful and ruptured tendons, rather than a uniform increase in all MMPs [78]. In cultured human tendon cells, IL‐1β increases the expression of selected matrix‐degrading enzymes, including MMP‐1, MMP‐3, MMP‐13, and ADAMTS‐4 [79]. Antioxidant therapy is considered a potential strategy for alleviating oxidative stress and improving tendinopathy symptoms [76]. Additionally, mitochondrial dysfunction is one of the primary causes of increased ROS production in tendinopathy. As the primary site of cellular energy production, mitochondrial dysfunction exacerbates ROS generation, leading to cellular damage and inflammatory responses. Research indicates that mitochondrial dysfunction is closely associated with the development of tendinopathy. Mitochondrial dysfunction not only leads to increased ROS but also disrupts cellular energy metabolism and signaling pathways, thereby accelerating the degeneration of tendon tissue [75].

The identification of mechanotransduction abnormalities suggests that mechanical signaling pathways may represent potential therapeutic targets. Patients characterized by excessive mechanical sensitivity may benefit from interventions aimed at optimizing mechanical loading, regulating mechanosensitive pathways, or inhibiting pathological fibrosis‐associated signaling.

#### 3.2.2 Inflammatory Response

The pathogenesis of tendinopathy involves complex inflammatory processes, characterized by the infiltration of various immune cells such as macrophages, T cells, neutrophils, and mast cells, alongside the activation of multiple pro‐inflammatory signaling pathways involving cytokines like the interleukin family and TNF‐α. These elements form the core of the inflammatory response.

Macrophages participate in the inflammatory response of tendinopathy. They accumulate during the healing process following tendon injury and regulate the inflammatory response and tissue repair by secreting cytokines and other signaling molecules [80]. Research indicates that activation of M1 macrophages is closely associated with poor tendon healing [81]. Notably, M2 macrophages are linked to anti‐inflammatory processes and tissue repair, promoting inflammation resolution and tissue regeneration by secreting anti‐inflammatory cytokines such as IL‐10 [82]. Modulating macrophage polarization significantly influences the severity of inflammatory responses and the efficacy of tissue repair; the balance between M1 and M2 macrophages (i.e., the M1/M2 ratio) represents a potential therapeutic target and prognostic biomarker in tendon pathologies [83]. A higher M1/M2 ratio is associated with poor healing outcomes and persistent inflammation, whereas shifting the balance toward M2 polarization promotes tissue repair. For instance, adipose‐derived mesenchymal stromal cells (ASCs) can improve tendon healing by inducing the transition of M1 macrophages to M2 macrophages, thereby reducing inflammatory cytokine levels [84]. The roles of T cells, neutrophils, and mast cells in chronic inflammation cannot be overlooked. T cells regulate macrophage polarization by releasing cytokines such as IFN‐γ, thereby influencing inflammatory progression and tissue repair capacity [85]. The role of Piezo1 in mechanical signal transduction has been demonstrated to promote neutrophil recruitment in fibroblasts by activating NFAT1 and upregulating LIF expression [86], thereby influencing tendinopathy progression through inflammatory regulation. Mast cells participate in inflammatory response modulation by releasing histamine and other mediators, playing a significant role in the pathophysiology of chronic tendinopathy [87].

Conversely, the role of multiple cytokines in the inflammatory response of tendinopathy cannot be underestimated. These cytokines mediate the pathophysiology of tendinopathy, promoting sustained inflammation and tissue damage. First among these is the interleukin family, with studies clearly establishing the damaging effects of IL‐1β, IL‐6, IL‐17, and IL‐23 on tendons. IL‐1β modulates inflammation; its overexpression not only causes tissue damage and exacerbates inflammation [88] but also accelerates tendon degradation by activating the NF‐κB signaling pathway and promoting matrix metalloproteinase (MMP) expression [89]. IL‐6 modulates the intensity and duration of inflammatory responses in tendinopathy through interactions with other cytokines, thereby promoting inflammation. In the chronic inflammatory state of tendinopathy, IL‐17's effects extend beyond inflammation; it also participates in tendon ossification and fibrosis processes, influencing TSPC function and extracellular matrix composition [90, 91]. Notably, IL‐17 synergizes with TNF‐α to influence tendinopathy progression not only through direct proinflammatory effects but also by amplifying the inflammatory response [92]. IL‐23 contributes to inflammation at tendon insertion sites. By acting on ROR‐γt+ CD3+CD4‐CD8‐ resident T cells at tendon insertion sites, IL‐23 activates the production of multiple inflammatory mediators, including IL‐6 and IL‐17, thereby exacerbating inflammation [93]. Furthermore, the activation of various proinflammatory signaling pathways is closely associated with the inflammatory response in tendinopathy [94, 95]. Activation of the NF‐κB and MAPK signaling pathways significantly promotes inflammation and exacerbates tendinopathy [96, 97, 98]. Abnormal activation of the JAK‐STAT signaling pathway not only intensifies inflammatory responses but also damages the normal tissue structure of tendons [99].

From a precision medicine perspective, inflammatory‐dominant tendinopathy may represent a distinct biological subgroup. Molecular indicators such as IL‐1β, TNF‐α, IL‐17, and macrophage polarization status may provide potential biomarkers for identifying patients who could benefit from immune‐modulatory approaches.

#### 3.2.3 Abnormal Angiogenesis and Innervation

Angiogenesis is a key process in the pathophysiology of tendinopathy. First, hypoxia‐inducible factor (HIF‐1α) is activated under hypoxic conditions, subsequently upregulating the expression of vascular endothelial growth factor (VEGF), which promotes angiogenesis [100]. This process can also be synergistically induced by mechanical loading and inflammatory factors, collectively leading to disordered vascular proliferation. As a key molecule in vascular proliferation during tendinopathy, VEGF further exacerbates ECM disruption and mechanical overload intolerance by promoting angiogenesis and nerve infiltration [101]. Moreover, HIF‐1α also promotes angiogenesis during wound healing [102].

Pathological mechanisms of tendinopathy also involve abnormal innervation, where increased release of glutamate and substance P (SP), along with nerve terminal infiltration and heightened pain sensitivity, are key factors. Studies indicate that nerve terminal infiltration following injury can amplify local inflammatory responses, thereby intensifying pain perception [103]. As a primary excitatory neurotransmitter, increased glutamate release is closely linked to pain signal transmission. In tendinopathy, excessive glutamate release may hyperexcite nerve endings, thereby heightening pain sensitivity [104]. SP, a neuropeptide, also serves as a core component in inflammation and pain within tendinopathy. On one hand, its release at nerve endings is closely associated with pain perception and transmission, particularly in chronic pain states. On the other hand, elevated SP levels are associated with cellular proliferation and angiogenesis in tendon tissue, potentially exacerbating pathological changes in tendinopathy [105]. The synergistic interaction between increased glutamate and SP release can jointly induce nerve terminal infiltration, forming a positive feedback mechanism that further intensifies pain. Finally, studies have revealed that altered expression of neurotrophic markers such as nerve growth factor (NGF) and calcitonin gene‐related peptide (CGRP) following tendon injury is closely associated with ECM remodeling [106]. This suggests that nerve ingrowth not only contributes to pain generation but may also exacerbate pathology by influencing ECM remodeling.

These findings suggest that vascular‐neural remodeling is not merely a consequence of tendon degeneration but may represent an independent pathological driver. Therefore, antiangiogenic or antineurogenic strategies may be particularly relevant for patients with vascular–neural dominant phenotypes.

#### 3.2.4 Degradation and Remodeling of the ECM

ECM is a complex, highly organized three‐dimensional network that plays a structural and functional role in tissue organization, remodeling, and the regulation of cellular processes. The degradation and remodeling of the ECM is a process of metabolic reprogramming. It refers to the systematic adjustment and transformation of a cell's metabolic patterns under specific physiological or pathological conditions, in order to adapt to changes in the external environment and meet its own needs for growth and differentiation. The metabolic reprogramming and microenvironmental disruption in tendinopathy constitute a complex process primarily involving tendon structural breakdown and extracellular matrix degradation.

In fatigued tendons, increased glycosaminoglycans modulate microscale mechanical properties and viscoelasticity, thereby affecting tendon repair capacity [107]. Furthermore, abnormal glycosaminoglycan metabolism is closely associated with tendinopathy progression, where pathological overaccumulation may exacerbate tendon structural and functional deterioration [108]. Altered matrix components represent a critical factor in studying metabolic reprogramming during tendinopathy. The tendon ECM constitutes a hierarchical structure composed of collagen fibrils, proteoglycans, and glycoproteins, which regulate tissue architecture, hydration, and mechanical properties [109]. The mechanisms underlying tendinopathy are closely linked to disturbances in ECM homeostasis. During the pathological progression of tendinopathy, the degradation and remodeling of the ECM are strictly regulated by a dynamic balance between matrix metalloproteinases (MMPs) and their endogenous inhibitors, tissue inhibitor of metalloproteinases (TIMPs). This balance undergoes characteristic changes across different disease stages, progressing from early adaptive remodeling to mid‐stage homeostasis imbalance and finally to late‐stage degenerative changes. The most abundant component in healthy tendons is type I collagen, which accounts for approximately 70%–80% of the dry weight of the tendon and about 95% of the total collagen in the tendon; the remaining 5% consists of type III and type V collagen [67]. These changes affect cellular function and tissue mechanical properties, particularly collagen dysregulation including a decrease in type I collagen, an increase in type III collagen, and alterations in cross‐linking patterns, which lead to a decline in mechanical properties [110, 111]. In tendinopathy, ECM abnormalities may not only diminish tissue mechanical properties and exacerbate pathological progression but also trigger inflammatory responses and fibrosis [112, 113].

ECM disruption represents a critical transition from reversible tendon adaptation to irreversible degeneration. Therapeutic strategies targeting MMP/TIMP imbalance, fibrosis regulation, and matrix regeneration may provide opportunities for disease modification rather than symptomatic relief.

### 3.3 Implications for Mechanism‐Based Therapies

Although the molecular pathways associated with tendon pathologies are still under investigation, identifying key pathological nodes may provide a theoretical basis for mechanism‐based interventions. For example, excessive mechanical sensing may indicate a need for load optimization and modulation of force transduction; an inflammation‐dominant phenotype may benefit from immunomodulatory strategies; and an extracellular matrix degradation phenotype may require interventions targeting matrix repair. Future research integrating biomarkers, imaging, and clinical phenotypes is needed to validate these stratified strategies.

## 4 Diagnosis and Assessment Techniques

Accurate diagnosis of tendinopathy remains challenging because clinical symptoms often overlap among different pathological conditions and biological subtypes. This section summarizes current diagnostic strategies from a multimodal perspective, including clinical assessment, imaging evaluation, and biomarker exploration. We first introduce commonly used clinical evaluation tools and functional scoring systems, followed by advances in structural and functional imaging techniques. Finally, we discuss emerging molecular biomarkers and differential diagnosis approaches that may contribute to future disease stratification and precision management.

### 4.1 Clinical Assessment Methods

Clinical evaluation methods are indispensable for the diagnosis and management of tendinopathy symptoms. They primarily include the assessment of symptoms and signs, imaging studies, and biomarker testing. These evaluation methods not only help clinicians identify various types of tendinopathy and better understand patients’ functional limitations and pain levels but also provide a basis for developing personalized treatment plans.

#### 4.1.1 Assessment of Symptoms and Signs

The assessment of symptoms and signs of tendinopathy includes morning stiffness, activity‐related pain, localized tenderness and swelling, and pain provocation tests. Morning stiffness and functional limitations are common manifestations of tendinopathy. Although functional limitations are often assessed through physical examinations, the results of these tests do not predict patient outcomes [114]; nevertheless, these tests remain important tools for evaluating the degree of functional limitation. Standardized pain maps help patients accurately describe the location of pain, thereby improving diagnostic accuracy [115]. The pathological features of tendinopathy include proteoglycan accumulation, swelling caused by fluid retention, and vascular proliferation, which can damage tissue and lead to persistent inflammation and pain [3]; assessing morning stiffness and activity‐related pain is crucial for determining disease severity and formulating a treatment plan. Local tenderness and swelling are typical signs of tendinopathy; these can be combined with physical examination and imaging studies (such as ultrasound and MRI) to observe changes in tendon structure and guide treatment decisions [116, 117]. Pain provocation tests (such as single‐leg squats and resistance knee extensions) demonstrate moderate‐to‐high sensitivity and specificity in the assessment of patellar tendinopathy [118]. The heel‐raise test and the Royal London Hospital test are commonly used methods for assessing the Achilles tendon; the former is diagnostically valuable for identifying tendon pain during single‐leg heel raises and jumping, while the latter evaluates sensitivity and specificity by assessing pain changes during weight‐bearing activities [119].

Functional assessment tools are indispensable in the management of tendinopathy, including the Victoria Institute of Sport's Achilles Tendon Assessment (VISA‐A) and Patellar Assessment (VISA‐P) scales, and the Oslo Sports Trauma Research Center (OSTRC) Overuse Injury Questionnaire.

The minimum clinically important difference (MCID) for the VISA‐A in patients with Achilles tendinopathy is 8–12 points (typically 8 points), while the MCID for the VISA‐P in patellar tendinopathy is approximately 6–8 points. Studies have confirmed the validity of the VISA‐A [120] and its good cross‐cultural validity [121, 122]; the VISA‐P is both sensitive and specific for patellar tendinopathy. The OSTRC can be used in conjunction with the VISA, while the TENDINS‐A outperforms the VISA‐A in the assessment of Achilles tendinopathy [123]. Psychological factors warrant consideration in the assessment of tendinopathy. Greater pain catastrophizing and depressive symptoms, together with lower pain self‐efficacy, are associated with more severe gluteal tendinopathy [124]. Nevertheless, evidence for psychological predictors of subsequent tendon‐related outcomes remains limited and inconsistent [125]. In addition, the patient‐reported outcomes measurement information system (PROMIS) physical function and pain interference short forms have been evaluated in midportion Achilles tendinopathy. In a longitudinal comparison, the VISA‐A was the most responsive measure assessed, indicating that outcome instruments should be selected for the specific population and measurement purpose [126].

In summary, the diverse assessment methods and tools described above provide reliable approaches for both clinical practice and research. When clinical presentations are atypical or standard treatments are ineffective, conditions such as paratendinopathy, bursitis, tendon rupture, enthesitis, nerve entrapment, or referred pain should be considered, and ultrasound or MRI is recommended for differential diagnosis.

#### 4.1.2 Imaging Diagnosis

The multifaceted application of imaging technologies—including commonly used ultrasound and magnetic resonance imaging (MRI), as well as emerging techniques such as shear‐wave elastography (SWE) and submicroscopic blood flow imaging (SMI)—is comprehensively revolutionizing the diagnosis and management of tendinopathy, ranging from structural imaging and functional assessment to early biomechanical detection. However, these methods also present challenges such as high costs and a heavy reliance on specialized personnel.

Ultrasound and MRI are the core imaging modalities for diagnosing tendinopathy. Ultrasound offers the advantages of bedside accessibility and dynamic assessment, allowing real‐time observation of tendon changes during various movements [127]. Typical findings include thickening, hypoechogenicity, collagen disorganization, and neovascularization. Thickening is associated with high‐intensity exercise, while hypoechogenicity suggests changes in internal structure [128]. Neovascularization may represent a pathological (persistent pain) or physiological response, and Doppler ultrasound aids in differentiation [129, 130, 131]. Ultrasound is not only used for diagnosis but also to guide treatment; for example, ultrasound‐guided electrocoagulation for refractory patellar tendinopathy yields good results with a low complication rate [132], and treatment efficacy can be monitored by assessing changes in thickness and blood flow [133]. MRI has distinct advantages for evaluating deep lesions and injuries at the tendon‐bone interface, and it is superior to ultrasound and X‐rays in assessing dysfunction of the posterior tibial tendon [134]. For tendon‐bone interface injuries, MRI can visualize fibrocartilage formation, which aids in assessing the quality of healing following rotator cuff repair [135]; T2‐weighted images can reveal abnormal ECM signals to identify degeneration and inflammation [136], and changes in signal intensity during postoperative evaluation can be used to assess healing status and guide rehabilitation [137]. However, MRI is costly, has limited accessibility, requires a long examination time, and is contraindicated for patients with metal implants, claustrophobia, or certain medical devices.

Among emerging imaging technologies, SWE can detect biomechanical changes in tendons before structural alterations occur, assess the extent of mechanical dysfunction by quantifying stiffness [138], and has the potential to evaluate lesion severity and predict healing [139]. Studies on the Achilles tendon have confirmed that the elastic modulus measured by SWE correlates highly with biomechanical findings [140] and can distinguish between healthy and pathological tendons. The use of shear wave velocity as an imaging biomarker [141] is of great significance for early diagnosis and monitoring [142]. SWE can also monitor changes in elasticity during healing [143] and, when combined with traditional functional scores, assess postoperative recovery [144]. However, its clinical adoption is limited by equipment availability, the need for specialized training, and high costs. SMI outperforms conventional Doppler in detecting tendon neovascularization, particularly in identifying new blood vessels in rotator cuff lesions, and shows a stronger correlation with pain and functional deficits [145]; it also aids in assessing the neovascularization and maturation status following anterior cruciate ligament tears [146]. Despite their advantages, the widespread clinical adoption of SWE and SMI is currently limited by equipment availability, the need for specialized training, and higher costs compared with conventional ultrasound. In summary, these emerging technologies do not replace conventional imaging but rather complement existing methods, improve the sensitivity of early diagnosis, and provide more precise guidance for clinical treatment.

#### 4.1.3 Biomarker Exploration

Biomarker testing is gaining increasing attention in the diagnosis of tendinopathy and is primarily divided into two categories: tissue biomarkers and body fluid biomarkers. Regarding tissue biomarkers, chronic inflammation (particularly in Achilles tendinopathy) is associated with the NF‐κB, interferon, and STAT‐6 pathways, as well as elevated expression of IL‐1β and IL‐33, which are linked to degeneration and reduced repair capacity [147]; MMP activity varies significantly across different subtypes, and elevated levels during the chronic phase can lead to excessive collagen degradation [148]; changes in the type III/type I collagen ratio are associated with decreased mechanical properties. Among body fluid biomarkers, IL‐6 is highly expressed in tenosynovitis [149, 150]; miR‐148a‐3p promotes angiogenesis by upregulating Tsp‐4 through targeting KLF6; miR‐140 and miR‐135 synergistically promote cellular reprogramming to aid regeneration [151, 152]; IL‐33 relieves the inhibition of type III collagen synthesis by downregulating miR‐29a, thereby promoting degenerative remodeling. However, no single biomarker has yet been validated for independent use in diagnosis or prognosis; their use is recommended only as an adjunct in specific research or clinical contexts (e.g., IL‐1β, IL‐6, and MMP‐3 to guide anti‐inflammatory therapy; MMP or collagen ratios to assess treatment efficacy; and genetic polymorphisms or microRNAs for risk stratification). Standardized testing and multicenter validation are required, and these biomarkers cannot replace clinical examinations and imaging. Accurate diagnosis of tendinopathy relies on a multimodal approach, including clinical evaluation (symptoms, functional testing, VISA‐A/P scales), imaging techniques (ultrasound, MRI, SWE, SMI), and tissue/body fluid biomarkers, the latter of which offer a promising avenue for early diagnosis and disease stratification.

### 4.2 Differential Diagnosis

Tendinopathy is a common disorder of the musculoskeletal system, but not all cases of pain or limited mobility are caused by degenerative tendinopathy. Therefore, differential diagnosis should be conducted by combining medical history, physical examination, and imaging findings [153], following the principles of “prioritizing location, stepwise exclusion, and imaging verification”. This article will discuss the differential diagnosis according to four categories: tendon‐related, osteoarticular, neurogenic, and muscular abnormalities (Figure 5).

**FIGURE 5:**
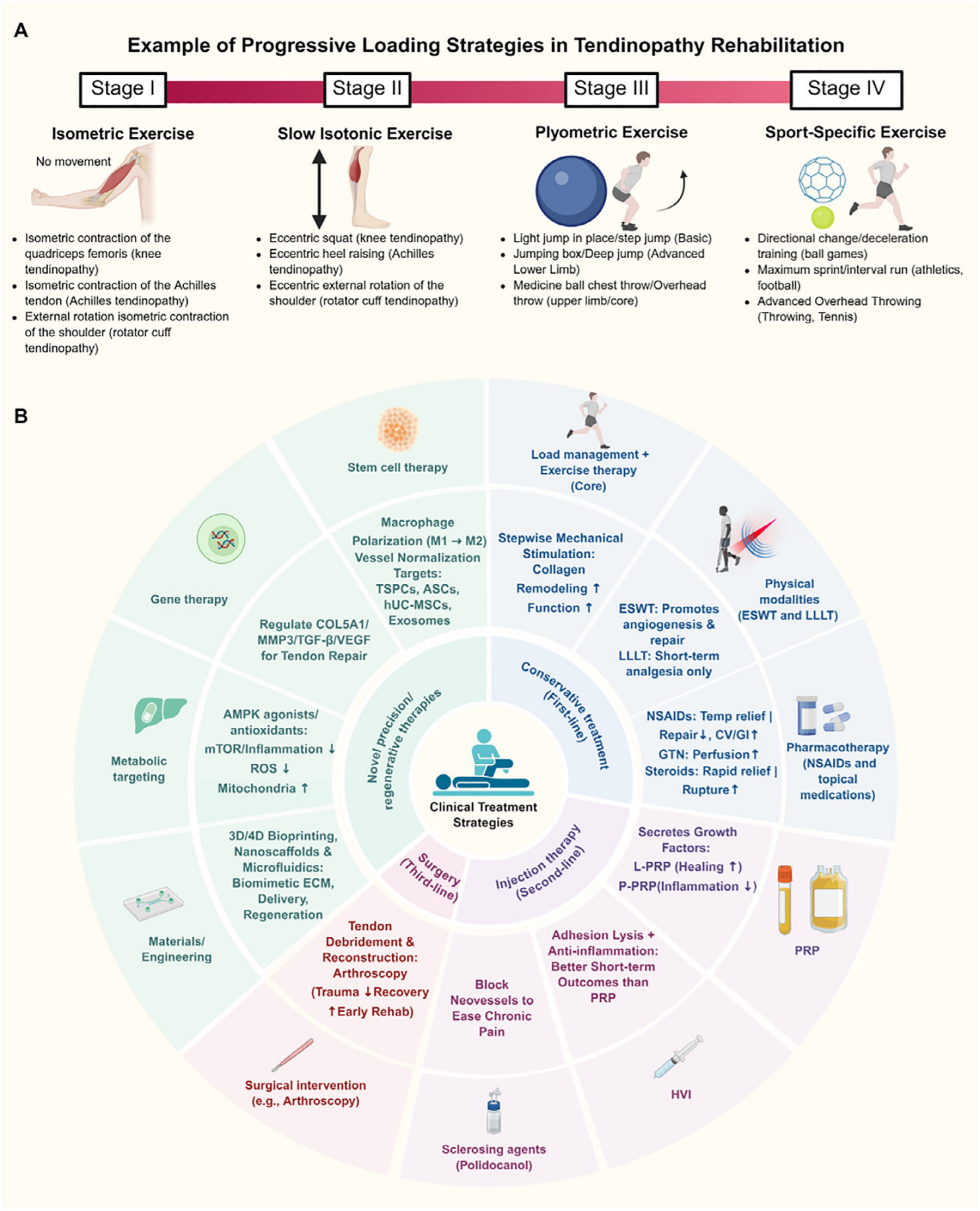
Differential Diagnosis of Tendinopathy. Not all pain in the Achilles tendon region is caused by degenerative tendinopathy; it is important to carefully distinguish between tendon‐related, osteoarticular, neurogenic, and muscular abnormalities.

#### 4.2.1 Tendon‐Related Lesions

These conditions center on degeneration or injury to the tendon itself, and pain may be triggered by isometric contractions. Insertional Achilles tendinopathy presents with pain at the insertion site on the posterior superior margin of the calcaneus; pain is particularly pronounced upon initial movement after waking or at rest, and direct pressure can provoke pain. Imaging reveals thickening, calcification, or osteophyte formation at the insertion site; care must be taken to differentiate this condition from Haglund's deformity and posterior calcaneal bursitis. Pain associated with mid‐calcaneal Achilles tendinopathy is located 2–6 cm above the insertion site. The affected tendon appears spindle‐shaped and swollen locally, with prominent load‐related pain (e.g., during jumping or running). The heel‐raise test is positive, and ultrasound or MRI may reveal thickening of the tendon body, hypoechoic areas, collagen disorganization, and neovascularization. Paratendinitis or partial tears present as sharp pain during movement in the paratendinous tissues or focal areas within the tendon; partial tears may be associated with a palpable “depression sign” or decreased heel‐raise strength. Imaging may reveal paratendinous edema or focal discontinuities. Pain associated with plantar tendonopathy is located on the medial side of the Achilles tendon or the posteromedial aspect of the lower leg; it presents as a deep, aching pain that worsens with ankle dorsiflexion and knee extension. MRI shows increased signal intensity along the course of the plantar tendon. Pain associated with retrocalcaneal tendon or flexor hallucis longus tendon pathologies is located behind the medial malleolus (medial aspect of the foot). It is triggered by prolonged walking or resistance to foot inversion. Imaging may reveal synovial effusion, thickening, or tears in the tendon sheath.

#### 4.2.2 Osteoarticular Lesions

Diseases of the joints and their associated structures can refer pain to the Achilles tendon region and must be thoroughly investigated. Haglund's deformity (posterior superior lateral calcaneal exostosis) causes pain on the posterior superior lateral aspect of the calcaneus; pressure from a hard heel causes “grinding pain.” A localized bony prominence is visible externally, often forming “Haglund's triad” (deformity + bursitis + Achilles tendinopathy). Posterior calcaneal bursitis causes pain deep within the space between the calcaneus and the Achilles tendon; severe pain occurs when the bursa is compressed during passive dorsiflexion of the ankle. MRI reveals bursal effusion and thickening; superficial bursitis, located subcutaneously behind the Achilles tendon, presents with redness, warmth, and swelling of the skin, often caused by friction from new shoes. Ultrasound shows a subcutaneous cystic anechoic area. Subtalar joint pain is located deep on the lateral side of the heel. Pain is significant when walking on uneven surfaces or during ankle inversion; joint compression triggers pain. MRI or CT scans may reveal narrowing of the joint space or signs of arthritis. Posterior ankle impingement syndrome, or talotriangular pain, is located deep in the posterior aspect of the ankle (behind the talus). Marked posterior tenderness is present during maximum plantar flexion (e.g., standing on tiptoes in ballet or running downhill). Lateral X‐rays may reveal the talotriangular process or Stieda's process, while MRI may show bone marrow edema or impingement of surrounding soft tissues. Calcaneal stress fracture presents with diffuse, deep, persistent pain at rest and nocturnal pain in the body of the calcaneus. The calcaneal compression test is positive; X‐rays may be negative in the early stages, while MRI or CT scans reveal bone edema and a fracture line. Sever's disease (calcaneal apophysitis) most commonly affects adolescents aged 8–15 years. Pain is located behind the calcaneal tuberosity, is severe after activity and relieved by rest, and the bilateral calcaneal compression test is positive. X‐rays show increased bone density in the calcaneus.

#### 4.2.3 Neurogenic Disorders

Approximately 10%–15% of pain in the Achilles tendon region is related to nerve compression, characterized by sensory abnormalities such as a burning sensation, numbness, or tingling. Pain associated with sural nerve lesions is located on the lateral aspect of the heel and the lateral edge of the foot, accompanied by a burning sensation or decreased sensation. It is often related to compression by the shoe upper or postoperative scar tissue. Tinel's sign (numbness induced by tapping along the nerve's course) is positive, and nerve ultrasound or electrophysiological studies may reveal slowed conduction velocity or the formation of a neuroma.

#### 4.2.4 Other Abnormal Lesions

Symptoms of the accessory soleus muscle manifest as a sensation of soreness and distension during physical activity in the area posterior and superior to the medial malleolus (anterior to the medial aspect of the Achilles tendon); a soft tissue mass (muscle variant) may be palpable, and symptoms worsen during resisted plantar flexion of the ankle. MRI may reveal an abnormal accessory muscle bundle on the medial aspect of the calcaneus. Calcaneal exostosis (non‐Haglund) causes pain beneath the calcaneus or at the medial/lateral bony prominences; pain results from compression of surrounding soft tissues by the bony protrusion, and X‐rays or CT scans reveal bony protrusions [154]. In addition, systemic diseases should be considered, such as seronegative spondyloarthropathies, familial hypercholesterolemia, endocrine or hormonal disorders, a history of fluoroquinolone use, diabetes, or metabolic syndrome; treatment strategies for tendon pathologies caused by these conditions differ significantly [155]. When clinical presentations are atypical or there is a poor response to conventional treatment, ultrasound or MRI should be actively utilized for differential diagnosis to avoid misdiagnosis as simple Achilles tendinopathy and subsequent delay in targeted treatment.

## 5 Precision Treatment Strategies Based on Biological Stratification

Currently, treatment for tendinopathies is primarily selected based on anatomical location, severity of symptoms, and the clinician's experience, and includes conservative treatment, interventional therapies, and regenerative treatment strategies (Figure 6). However, some studies suggest that, due to potential biological heterogeneity, treatment responses may vary significantly among patients. Future treatment strategies may need to be stratified according to the underlying pathological mechanisms, rather than solely based on anatomical location (Table 3). Although numerous regenerative strategies have emerged, their clinical translation remains limited because biological subtypes have not yet been adequately defined.

**FIGURE 6:**
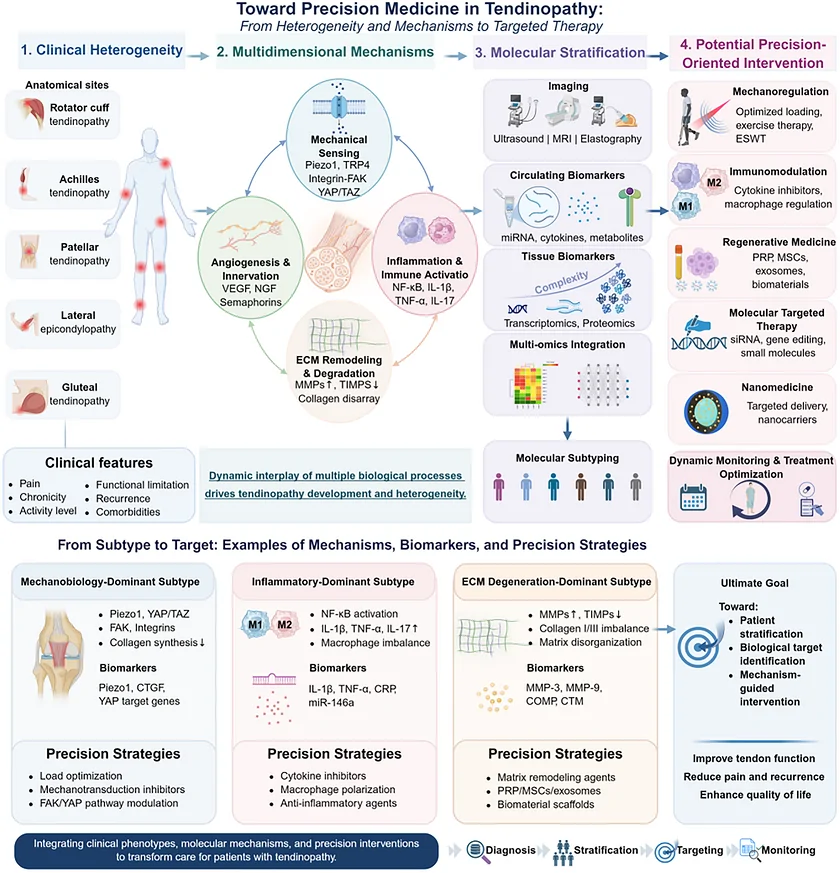
Treatment strategies for tendinopathy. (A) Targeted training for four different stages. (B) The treatment of tendinopathy has evolved from simple symptom relief to a series of tiered treatment options, including conservative treatment, injection therapy, surgical intervention, and comprehensive treatment strategies such as stem cell therapy and nanotechnology.

**TABLE 3:** Targeted therapeutic strategies for tendinopathy.

Therapy category			Main effect	Notes/Limitations
Mechanobiology‐targeted therapy	Load management and exercise therapy		Progressive load modulation; phased isometric/eccentric/sport‑specific training [156, 157, 158, 159, 160, 161, 162, 163, 164, 165]	Requires adherence; risk of recurrence if loaded prematurely
	Physical Therapy	ESWT	reduces inflammation, stimulates collagen synthesis and cell proliferation to improve pain and function [167, 168, 169, 170, 171, 172, 173]	High‑energy (≥0.28 mJ/mm^2^) for calcific tendinopathy; low‑energy (≤0.12 mJ/mm^2^) for analgesia
		LLLT	Low‑level laser therapy (especially 904 nm wavelength): provides short‑term pain relief [174, 175, 176, 177, 178]	Efficacy debated; some studies show no superiority over placebo or conventional treatment
		Adjunctive	Ultrasound, electrical stimulation, sensorimotor training (SMT) [179, 180, 181]	Evidence quality is low; SMT may improve function but requires further validation
	Surgical treatment		Debridement, repair or reconstruction; removes degenerated tissue and restores continuity [182, 183, 184, 185, 186, 187, 188, 189, 190, 191]	Reserved for refractory cases; risk of complications; non‑inferior to conservative care in some sites
Immunomodulatory therapy	Pharmaco‐therapy	NSAIDs	short‑term analgesia [192, 193]	Long‑term use risks renal and cardiovascular, GI events
		corticosteroids	rapid short‑term pain relief [12, 171, 194]	Long‑term use increases tendon rupture risk; injection frequency must be strictly limited
	Injection Therapy	PRP	L‑PRP promotes early repair, P‑PRP reduces inflammation and promotes matrix synthesis [195, 196, 197, 198, 199, 200, 201, 202, 203, 204, 205]	efficacy varies by lesion type
		HVI	Disrupts adhesions and neovascularization; flushes inflammatory mediators [206, 207, 208, 209]	Long‑term use may lead to tendon degeneration and damage
ECM remodeling	Stem cell therapy	TSPCs	differentiate into tenocytes and secrete exosomes to promote repair [57, 210, 211]	Long‑term safety and efficacy require further validation
		ASCs	Secretes anti‑inflammatory and pro‑regenerative factors; enhances ECM organization [212, 213, 214, 215]	Short‑term improvements in pain and function; long‑term data are lacking
		hUC‐MSCs	inhibit endothelial proliferation and reduce inflammation [216]	Safety assessment remains insufficient
	DNA hydrogels		Act as an artificial ECM to prolong stem cell retention within the tendon [217]	Enhances healing outcomes; long‑term safety unknown
	Nanotechnology and tissue engineering		Mimics ECM architecture; enables targeted delivery and controlled release of therapeutic agents [57, 220, 221]	Biocompatibility and long‑term effects require further investigation
	Sclerosing agents		reduce abnormal angiogenesis, alleviate short‑term discomfort [222]	Primarily used for chronic Achilles tendinopathy
	Gene therapy		Modulate COL5A1/MMP3 expression; deliver growth factors such as TGF‑β and VEGF [30, 223, 224, 225, 226]	Targets genetic susceptibility and tissue repair; currently at the research stage
	Nitroglycerin patches		releases nitric oxide to improve local blood flow and promote healing [227, 228]	High incidence of headache
	Antioxidant therapy		Scavenges ROS; restores redox balance; protects against oxidative injury [229]	Improves tenocyte function; optimal regimens remain to be established

### 5.1 Mechanobiology‐Targeted Therapy

#### 5.1.1 Load Management and Exercise Therapy

Load management combined with exercise therapy is the most used clinical approach for tendinopathy; combining pain education with exercise can significantly improve pain and function in patients with chronic Achilles tendinopathy [156]. Reducing compressive load on the tendon can effectively alleviate symptoms [157], and personalized load management protocols can better accommodate a patient's biomechanical characteristics [158], not only relieving symptoms but also preventing recurrence [159]. Future efforts should focus on further optimizing individualized load management protocols [160].

Exercise therapy is another common and effective treatment approach, centered on gradually increasing load to promote mechanical adaptation of the tendon and ECM remodeling [161]. Rehabilitation should be conducted in phases: early isometric training can enhance muscle strength without increasing tendon load [162]; in the second phase, slow isometric training (such as heel raises) improves tendon tolerance [163]; in the third phase, strengthening training enhances functional performance and reduces the risk of re‐injury [164]; and in the fourth phase, sport‐specific training helps patients return to competition [163, 164, 165].

#### 5.1.2 Physical Therapy and Physical Agent Therapy

Physical therapy is of paramount importance in alleviating pain and promoting healing [166], primarily including extracorporeal shock wave therapy (ESWT) and low‐level laser therapy (LLLT).

ESWT improves pain and function by promoting angiogenesis (upregulating VEGF) and tissue repair (reducing inflammation, promoting collagen synthesis, and stimulating cell proliferation) [167, 168, 169, 170, 171]. The efficacy of ESWT is related to energy levels: high‐energy ESWT (≥0.28 mJ/mm^2^) is more effective in improving function and resorbing calcifications, while low‐energy ESWT (≤0.12 mJ/mm^2^) tends to be more effective in relieving pain [172, 173]. LLLT (particularly at a wavelength of 904 nm) can significantly relieve short‐term pain associated with elbow tendinopathy [174] and nonspecific chronic lower back pain [175]; however, its efficacy remains controversial, as some studies have failed to demonstrate its superiority over placebo or conventional treatment [176, 177]. Efficacy may be related to the number of treatment sessions [178]. The evidence for adjunctive therapies such as ultrasound and electrical stimulation is of lower quality. Laser therapy is superior to ultrasound in improving myofascial pain in the shoulder [179]; electrical stimulation has also shown efficacy in certain cases, but its effectiveness relative to LLLT requires further validation [180]; sensorimotor training (SMT) can enhance function and relieve pain, but the evidence remains insufficient [181].

#### 5.1.3 Surgical Treatments

As a treatment that balances efficacy and risk, surgical intervention is indicated for patients in whom conservative treatment has failed after more than 12 months and who have severe functional impairment or are at risk of tendon rupture. Surgery for partial Achilles tendon tears yields good results [182, 183]; reconstruction of the patellar tendon following a tear can significantly improve function [184], with a postoperative return‐to‐sport rate as high as 89.8% [185]; arthroscopic surgery results in fewer complications and faster recovery compared with open surgery [186, 187], and both postoperative pain and functional scores show significant improvement during follow‐up [188, 189]. However, in cases of elbow tendinopathy and rotator cuff tendinopathy, there is no significant difference in efficacy between surgical and nonsurgical treatments [190, 191]. In summary, surgery should be selected with caution, and nonsurgical options should be thoroughly evaluated.

### 5.2 Immunomodulatory Therapy

#### 5.2.1 Pharmacological Treatment

Pharmacological treatment for tendinopathy typically includes oral medications such as nonsteroidal anti‐inflammatory drugs (NSAIDs), and topical treatment such as corticosteroids. NSAIDs are highly effective for pain relief in acute soft tissue injuries [192], but long‐term use can inhibit muscle regeneration and affect myocyte migration [193]. Topical corticosteroids provide good short‐term pain relief, but long‐term use may increase the risk of tendon rupture [171, 194]. In our view, topical medications should be selected with caution based on the patient's specific condition and treatment goals to maximize efficacy while minimizing risks.

#### 5.2.2 Injection Therapies

Common injection therapies currently used clinically for tendinopathy include platelet‐rich plasma (PRP) therapy, high‐volume injection (HVI) therapy, and emerging injectable treatments, with significant variations in their clinical efficacy.

Although PRP is more effective than traditional corticosteroid injections, its long‐term efficacy requires further validation [195, 196, 197]. While PRP injections show better medium‐ to long‐term outcomes than ESWT for patellar tendinopathy [198], some studies indicate no significant difference in efficacy compared with placebo [199, 200]. Leukocyte‐rich PRP (L‐PRP) promotes early tendon repair [201] but may hinder tendon healing in the treatment of tendon injuries [202, 203]. Leukocyte‐poor PRP (P‐PRP) offers greater advantages in reducing inflammation and promoting matrix synthesis [204]. The efficacy of PRP varies depending on the type of lesion, and future efforts should focus on standardizing preparation methods and application strategies [205]. HVI provides rapid symptom relief in the short term and is superior to PRP [206]; when combined with glucocorticoids, it can significantly improve function and relieve pain within 6–12 weeks [207, 208], but long‐term use may lead to tendon degeneration and damage [209].

### 5.3 ECM Remodeling

Stem cell therapy is currently one of the most actively researched fields. Tendon stem/progenitor cells (TSPCs) possess the ability to form colonies, differentiate in multiple directions, and self‐renew, making them an important cellular source for tendon repair. Exosomes derived from TSPCs (TSPC‐Exos) can promote healing at the tendon‐bone interface, modulate inflammation, and improve biomechanical properties [210]; when used in combination with tendon‐derived decellularized ECM hydrogels, they can induce M2 macrophage polarization, enhancing anti‐inflammatory effects and regenerative capacity [211]. Adipose‐derived mesenchymal stem cells (ASCs) promote tendon repair by secreting cytokines and regulating inflammation; when combined with growth factors, they can further improve tissue structure and mechanical properties [212, 213, 214]. Clinical studies show that treatment with TSPCs and ASCs can improve pain and function in the short term [215], but long‐term efficacy and safety still need to be verified. Engineered stem cell therapies derived from human umbilical cord mesenchymal stem cells (hUC‐MSCs) alleviate tendinopathy by inhibiting vascular endothelial proliferation and reducing inflammation [126]. DNA hydrogels, acting as artificial ECMs, can prolong the retention time of stem cells within tendons and enhance healing outcomes [217]. Overall, these emerging stem cell therapies offer new strategies by inhibiting excessive vascularization and promoting innervation; however, long‐term safety assessments are still lacking [218, 219].

Nanotechnology and tissue engineering provide a new materials science foundation for tendon repair. Nanostructured scaffolds can mimic the tendon ECM microenvironment, enabling targeted delivery and sustained release of cells or drugs to promote tendon regeneration and repair [57]. Inspired by the highly ordered nanotopological structure of rotator cuff tendons, analyzing molecular differences between healthy and injured tendons enables the design of more precise repair strategies that utilize biomaterial scaffolds to activate endogenous repair systems [220]. For example, nanotopological scaffolds can regulate the adhesion, proliferation, and differentiation of tendon‐derived cells, thereby aiding tendon repair [221]. Sclerosing agent injections (such as polidocanol) alleviate short‐term discomfort and accelerate functional recovery by reducing abnormal angiogenesis [222].

Gene therapy is another emerging field of research, aiming to improve tendon repair by regulating the expression of specific genes or delivering growth factors [223]. Variants in the COL5A1 and MMP3 genes are associated with the risk of Achilles tendinopathy, and there is an interactive effect between the two [224]. Furthermore, TGF‐β and VEGF serve as key regulators in tissue repair [225, 226], providing potential targets for gene‐targeted therapies.

Nitroglycerin patches improve blood flow by releasing nitric oxide and are effective for conditions such as shoulder impingement syndrome [227, 228], but they are associated with a high incidence of headaches. Antioxidants mitigate oxidative damage by scavenging ROS, thereby improving tendon cell function [229].

## 6 Challenges and Future Perspectives

Although substantial progress has been made in the study of tendinopathy, translating insights into effective clinical practice remains fraught with multiple challenges. This review systematically outlines current research gaps and future directions in three areas: basic research, diagnosis, and treatment.

### 6.1 Current Research Gaps

In the field of basic research on tendinopathy, the most critical task is to elucidate the core molecular mechanisms underlying its pathogenesis. Currently, the interactions among mechanical stress, inflammation, angiogenesis, and ECM remodeling remain incompletely understood. Mechanical loading can trigger tenocytes to release extracellular mitochondrial particles, induce macrophage chemotaxis, and increase the production of pro‐inflammatory factors [76], thereby affecting tendon structure and function by regulating collagen synthesis and degradation in tenocytes [230]. Various inflammatory mediators, such as fatty acid‐binding protein 4 (FABP4), prostaglandins, and lipoxins, are present in tendinopathy and are indispensable for regulating tendon inflammation and the ECM [231, 232]. Notably, different subtypes of tendinopathy exhibit heterogeneity in terms of angiogenesis and inflammatory characteristics, which may influence treatment outcomes [233]. Finally, further clarification is needed regarding whether sex differences in animals affect the mechanisms of tendinopathy, as suggested by basic research [234].

In terms of diagnosis, the greatest challenge lies in the lack of early biomarkers with high sensitivity and specificity, making it difficult to distinguish between inflammatory and degenerative subtypes. Specific molecular pathways, such as the IL‐17A pathway, may be involved in the early stages of tendinopathy, providing potential targets for early diagnosis and treatment [235]. The high prevalence of tendon abnormalities among asymptomatic athletes suggests that these abnormalities may be precursors to symptomatic tendinopathy, but the optimal timing for intervention remains unclear [236, 237]. Although bioinformatics and machine learning have identified potential early‐diagnostic genes such as MACROD1, these findings require clinical validation [238].

In terms of treatment, current intervention strategies, such as NSAIDs and corticosteroid injections, remain focused on symptom relief rather than reversing degenerative changes [239, 240], and there is a lack of precision intervention strategies based on individual genetic and metabolic characteristics [241]. Although stem cell therapy has shown promise [241, 242], the lack of personalized treatment plans continues to limit its efficacy. And mRNA‐based anti‐inflammatory strategies have demonstrated the potential to suppress inflammation and improve tendon structure in mouse models, offering a new direction for future treatments [243].

### 6.2 Key Research Directions

#### 6.2.1 Priorities in Basic Research

Basic research on tendinopathy focuses on validating molecular targets, particularly the regulatory roles and intervention potential of key molecules such as IL‐17, IL‐33, HIF‐1α, and Piezo1 in tendinopathy. The IL‐17 family is associated with bone healing, tendinous ossification, and aging; inhibition of IL‐17A reduces pathological bone formation [90, 244]. Dysregulation of IL‐33 is a key factor in tendon healing failure [245]. HIF‐1α promotes tendon cell differentiation and regeneration under hypoxic conditions; its inhibition reduces ectopic ossification [246, 247]. Under hyperglycemic conditions, HIF‐1α further enhances collagen synthesis by promoting the expression of Sox9 and Scleraxis, thereby protecting tendon cells from oxidative stress [248]. Piezo1, as a mechanosensitive channel, is widely involved in the musculoskeletal system, making it a potential therapeutic target [249]; changes in its activity significantly affect the mechanical properties of tendons [70, 250]. If possible, future research should use human tendon samples whenever possible to provide direct evidence. Multi‐omics integration not only holds promise for revealing hierarchical information underlying disease mechanisms but also facilitates the identification of disease‐related imaging and biological biomarkers [251]

The “immune‐metabolic‐neural” network is the central regulatory axis of the tendon microenvironment. In the immune component, hypoxia‐induced upregulation of HIF‐1α promotes the production of pro‐inflammatory factors and type III collagen synthesis [252]. The polarization state of macrophages (M1/M2 balance) significantly influences inflammatory and reparative outcomes [253, 254]. Excessive mechanical loading induces the release of mitochondrial particles, triggering an immune response. In the subsequent metabolic phase, metabolites (lactic acid, ROS) contribute to the sensitization of nerve endings [255]. Neuropeptides such as neuropeptide Y (NPY), SP, and CGRP, in turn, influence tendon repair by regulating angiogenesis and local immunity [256]. Future research should focus on investigating the interactions among the immune, metabolic, and neural systems in tendinopathy.

Optimizing animal models is also a key area in basic research. Mechanical loading can influence tendon health by modulating biomechanical properties and cellular responses [257]. In small animal models, high‐load mechanical stimulation can induce pathological changes similar to those seen in human tendon pathologies, such as collagen fiber rearrangement and ECM alterations [68]. In future research, treadmill‐induced disease models can be employed not only to accurately reproduce the pathological features of human tendon disorders, including cell proliferation, disorganized collagen fiber arrangement, and inflammatory responses [258], but also to study the biological responses of tendons under different loading conditions by adjusting running intensity and frequency [257]. This model can be combined with synchrotron micro‐CT to observe collagen rearrangement and damage propagation in real time under load [259], which will help advance the clinical treatment of tendinopathy and optimize rehabilitation strategies [260].

#### 6.2.2 Clinical Management and Trial Strategies

The future management of tendinopathy requires a paradigm shift from symptom‐based treatment toward mechanism‐guided precision medicine. This transition will depend on the development of reliable biomarkers, molecular classification systems, artificial intelligence‐assisted imaging analysis, and clinically validated targeted therapies.

The core of early intervention for tendinopathy is the development of prevention strategies based on risk factor screening. Genetic testing can identify certain single‐nucleotide polymorphisms (SNPs) to predict the risk of developing the condition [261]. Ultrasound evaluation can effectively detect morphological and biomechanical abnormalities in tendons, aiding in the identification of high‐risk individuals [262, 263]. Regarding preventive strategies, targeted exercise training is considered an effective method for reducing the incidence of tendinopathy [264]. At the same time, personalized exercise prescriptions can be adjusted based on tendon strain to optimize training outcomes and reduce the risk of injury [265]. The combination of these three approaches can significantly reduce the incidence of tendinopathy in athletes, and strategies for cross‐population application can be further optimized in the future [266].

Clinical treatment of tendinopathy should be based on subtypes (such as inflammation‐dominant or degeneration‐dominant) to develop individualized strategies. For early‐stage inflammatory tendinopathy, anti‐inflammatory medications are crucial [231]. For degeneration‐dominant tendinopathy, TSPC therapy can promote tendon regeneration and healing [267], although results regarding imaging and biomechanical outcomes remain inconsistent [268]. Although combining anti‐inflammatory and regenerative therapies may enhance treatment outcomes [269], evidence regarding efficacy remains inconsistent. Future methods and processes for standardized outcome assessment will help objectively evaluate the effectiveness of precision treatments.

Clinical trials remain an indispensable component of advancing diagnosis and treatment, with a focus on large‐scale randomized controlled trials (RCTs) that standardize outcome measures (pain, function, structure, and quality of life) and include long‐term follow‐up. The ICON 2019 Consensus defines nine core domains to standardize outcome reporting [270], with pain and functional assessment serving as key indicators; exercise load significantly influences outcomes [271]. Studies indicate that combining different treatment modalities (such as PRP and ESWT) can accelerate tendon pain relief and functional improvement [272]. Therefore, long‐term RCTs are needed to verify the durability of efficacy and safety [273], and the long‐term benefits of different treatment regimens should be considered when designing such trials [274]. It is worth noting that, due to a lack of standardization, different centers may encounter issues regarding sample collection, processing, and data analysis procedures, leading to poor reproducibility. These issues need to be gradually resolved through long‐term clinical practice.

#### 6.2.3 Technological Innovation and Translation

Significant progress has been made in recent years in diagnostic technologies for tendinopathy, primarily due to the rapid development of wearable devices and artificial intelligence (AI). Wearable devices utilizing shear‐wave strain measurement technology can assess tendon loading patterns in real time [275]; elastography can identify tendon lesions before abnormalities are detected by conventional ultrasound [276]. AI integrated with muscle MRI enables quantitative analysis and improves disease prediction [277]; photoacoustic imaging (PAI) allows for noninvasive detection of inflammatory areas [278]. The application of AI in ultrasound image analysis significantly improves diagnostic accuracy and enables automatic lesion recognition [279]. Combining AI with wearable devices allows for precise monitoring of joint torque, prediction of Achilles tendon load, and optimization of rehabilitation strategies [280, 281, 282, 283, 284, 285, 286]. Although these technologies hold great promise, the clinical translation of AI and wearable devices remains in the research phase. Future efforts should focus on strengthening technical research and clinical validation, including multicenter datasets, the detection of hidden human injuries, and real‐world accuracy testing of wearable devices.

In terms of therapeutic technologies, 3D bioprinting and microfluidics offer new approaches for constructing tendon substitutes and enabling targeted drug delivery [287]; 4D printing, in turn, drives the development of personalized implants [288]. However, the development and research of these new materials, as well as new methods for targeted drug delivery, face significant challenges regarding safety and efficacy when transitioning from animal studies to clinical applications. Ensuring that the effects observed in animal experiments can be replicated in humans requires not only long‐term human trials, which involve numerous ethical issues, but also the demonstration of efficacy superior to existing therapies, while ensuring adequate safety, such as verifying that new materials do not cause harm to the human body. Every stage of this process requires the long‐term collaborative efforts of researchers, clinicians, and patients.

## 7 Conclusion and Outlook

Tendinopathy is a complex disease driven by dynamic interactions among mechanical, inflammatory, vascular, neural, and metabolic signals; its pathological essence lies in an imbalance in tendon homeostasis and impaired repair. The core mechanism, the “multidimensional tendinopathy remodeling network”, is initiated by excessive mechanical loading. It is transduced via force‐sensitive ion channels such as Piezo1 and TRPV4, triggering calcium influx, mitochondrial ROS production, and downstream pro‐inflammatory signaling. This, in turn, mediates the recruitment of immune cells through chemokines (CCL2, CXCL1, etc.) and their receptors, forming a self‐reinforcing loop of inflammation, pain, and matrix degradation. During this process, the HIF‐1α/VEGF pathway drives abnormal angiogenesis and nerve ingrowth, while extracellular vesicles (EVs) carrying signaling molecules such as miR‐29a and IL‐33 transmit pathological information between the tendon and muscle, mediating secondary muscle atrophy and chronic pain. It is worth noting that different tendinopathy subtypes exhibit significant heterogeneity in terms of inflammatory dominance, degree of vascularization, and matrix remodeling characteristics. This necessitates that our understanding of the “multidimensional tendinopathy remodeling network” should not be limited to a single pathway but should shift toward a systems‐level perspective that integrates mechanical, immune, metabolic, and neural interactions. This shift in understanding is central to the evolution of tendinopathy research from a descriptive observational approach to a mechanism‐driven paradigm. By integrating multi‐omics data with artificial intelligence (such as AI‐driven protein structure prediction models like AlphaFold‐2), researchers can gain a more precise understanding of the pathological mechanisms underlying tendinopathy, thereby developing more targeted and effective treatment strategies [289, 290].

The current diagnostic framework has evolved from a single‐parameter assessment to a multimodal integrated approach, encompassing the synergistic application of clinical scales (VISA‐A/VISA‐P), imaging techniques (ultrasound, MRI, SWE, SMI), and biomarkers (inflammatory cytokines, MMPs, microRNAs). However, existing methods still struggle to distinguish between inflammation‐dominant and degeneration‐dominant types in the early stages, and there is a lack of widely validated diagnostic biomarkers. The introduction of AI‐assisted image analysis and wearable devices has provided new technical pathways for dynamically monitoring tendon mechanical properties and identifying early‐stage lesions. Building on this foundation, a multi‐omics integration strategy combining genomics, transcriptomics, and metabolomics holds promise for identifying molecular signature sets capable of distinguishing between subtypes, thereby breaking through the current diagnostic impasse of “disease heterogeneity.” This trend reflects the deep penetration of the precision medicine concept into the field of tendon disease diagnosis.

Current therapeutic strategies for tendinopathy are mainly based on anatomical location and symptom severity rather than underlying biological characteristics. The multidimensional tendinopathy remodeling network suggests that patients with clinically similar symptoms may represent biologically distinct subgroups requiring different therapeutic approaches. For example, patients dominated by mechanotransduction abnormalities may benefit from interventions targeting mechanical signaling pathways, whereas individuals exhibiting inflammatory signatures may require immune‐modulatory strategies. Similarly, patients with ECM‐dominant degeneration or metabolic dysfunction may represent distinct therapeutic populations. The key to optimizing future treatments lies in establishing a precision stratification system based on molecular subtyping and promoting the seamless integration of targeted drugs with regenerative strategies. At the same time, patient involvement in study design and interdisciplinary team collaboration are increasingly recognized as crucial, helping to advance personalized treatment for tendinopathy.

Despite substantial advances in understanding tendinopathy biology, translation of molecular discoveries into clinical practice remains limited. The major challenge is not the absence of potential therapeutic targets, but rather the lack of biologically meaningful patient classification systems that can guide treatment selection. We propose that future tendinopathy management should transition from an empiric symptom‐based approach toward precision medicine integrating clinical phenotyping, molecular biomarkers, advanced imaging, and targeted intervention. Such a paradigm shift may enable identification of disease‐driving mechanisms in individual patients and facilitate the development of truly disease‐modifying therapies.

## Author Contributions

Yang Wu: conceptualization and writing – review and editing. Junyi Long: writing – review and editing and writing – original draft. Meng Yang: conceptualization. Hongjie Huang: funding acquisition and resources. Jianquan Wang: funding acquisition and resources. All authors approved the final version of the manuscript.

## Funding

This work was supported by the Peking University Third Hospital “Bocai Hebi” Program; the National Natural Science Foundation of China (Grant Nos. 22674008 and 82372418); the China Postdoctoral Science Foundation (Grant No. 2026M794242); and the Capital's Funds for Health Improvement and Research (CFH) (Grant No. 2026‐2‐4094).

## Ethics Statement

The authors have nothing to report.

## Conflicts of Interest

The authors declare no conflicts of interest.

## Data Availability

The authors have nothing to report.

## References

[1] N. L. Millar , K. G. Silbernagel , K. Thorborg , et al., “Tendinopathy,” Nature Reviews Disease Primers 7, no. 1 (2021): 1. 10.1038/s41572-020-00234-1 33414454

[2] A. Bedi , J. Bishop , J. Keener , et al., “Rotator Cuff Tears,” Nature Reviews Disease Primers 10, no. 1 (2024): 8. 10.1038/s41572-024-00492-3 38332156

[3] A. Traweger , A. Scott , M. Kjaer , et al., “Achilles Tendinopathy,” Nature Reviews Disease Primers 11, no. 1 (2025): 20. 10.1038/s41572-025-00602-9 40148342

[4] J. A. Peters , J. Zwerver , R. L. Diercks , et al., “Preventive Interventions for Tendinopathy: A Systematic Review,” Journal of Science and Medicine in Sport 19, no. 3 (2016): 205–211. 10.1016/j.jsams.2015.03.008 25981200

[5] L. R. Lopes , D. E. Machado , R. A. Goes , et al., “Implications of Tendinopathy on the Sports Career: Epidemiological and Clinical Profile of High‐Performance Athletes,” Physical Therapy in Sport 72 (2025): 59–68. 10.1016/j.ptsp.2025.02.003 39951859

[6] L. C. Slane , F. Dandois , S. Bogaerts , et al., “Non‐uniformity in the Healthy Patellar Tendon Is Greater in Males and Similar in Different Age Groups,” Journal of Biomechanics 80 (2018): 16–22. 10.1016/j.jbiomech.2018.08.021 PMC6231227 30224164

[7] A. Giai Via , F. Oliva , and J. Padulo , “Insertional Calcific Tendinopathy of the Achilles Tendon and Dysmetabolic Diseases: An Epidemiological Survey,” Clinical Journal of Sport Medicine 32, no. 1 (2022): e68–e73. 10.1097/JSM.0000000000000881 32976122

[8] S. R. Filbay , S. T. Skou , G. S. Bullock , et al., “Long‐term Quality of Life, Work Limitation, Physical Activity, Economic Cost and Disease Burden Following ACL and Meniscal Injury: A Systematic Review and Meta‐Analysis for the OPTIKNEE Consensus,” British Journal of Sports Medicine 56, no. 24 (2022): 1465–1474. 10.1136/bjsports-2022-105626 36171078

[9] T. S. O. Sleeswijk Visser , A. C. van der Vlist , R. F. van Oosterom , et al., “Impact of Chronic Achilles Tendinopathy on Health‐related Quality of Life, Work Performance, Healthcare Utilisation and Costs,” BMJ Open Sport & Exercise Medicine 7, no. 1 (2021): e001023. 10.1136/bmjsem-2020-001023 PMC8006822 33868707

[10] B. Roets , H. Abrahamse , and A. Crous , “Biomaterial Properties and Differentiation Strategies for Tenogenic Differentiation of Mesenchymal Stem Cells,” Cells 14, no. 6 (2025): 452. 10.3390/cells14060452 PMC11940850 40136701

[11] I. Burton , “Autoregulation in Resistance Training for Lower Limb Tendinopathy: A Potential Method for Addressing Individual Factors, Intervention Issues, and Inadequate Outcomes,” Frontiers in Physiology 12 (2021): 704306. 10.3389/fphys.2021.704306 PMC8375597 34421641

[12] S. F. Kane , L. H. Olewinski , and K. S. Tamminga , “Management of Chronic Tendon Injuries,” American Family Physician 100, no. 3 (2019): 147–157. 31361101

[13] M. Cassel , H. Baur , A. Hirschmüller , et al., “Prevalence of Achilles and Patellar Tendinopathy and Their Association to Intratendinous Changes in Adolescent Athletes,” Scandinavian Journal of Medicine & Science in Sports 25, no. 3 (2015): e310–e318. 10.1111/sms.12318 25212527

[14] V. K. Morya , H. Shahid , J. Lang , et al., “Advancements in Therapeutic Approaches for Degenerative Tendinopathy: Evaluating Efficacy and Challenges,” International Journal of Molecular Sciences 25, no. 21 (2024 Nov 4): 11846. 10.3390/ijms252111846 PMC11545934 39519397

[15] K. K. L. Lau , J. P. Ng , S. K. K. Ling , et al., “Incidence Rates of Tendinopathies and Non‐traumatic Tendon Ruptures in Hong Kong: A 25‐Year Epidemiological Study Using Big Data From Public Hospitals,” Scientific Reports 16, no. 1 (2026): 3509. 10.1038/s41598-025-33465-x PMC12847841 41526611

[16] K. Walker‐Bone , K. T. Palmer , I. Reading , et al., “Occupation and Epicondylitis: A Population‐Based Study,” Rheumatology (Oxford) 51, no. 2 (2012): 305–310. 10.1093/rheumatology/ker228 PMC3427015 22019808

[17] R. G. Alrabaa , M. H. Lobao , and W. N. Levine , “Rotator Cuff Injuries in Tennis Players,” Current Reviews in Musculoskeletal Medicine 13, no. 6 (2020): 734–747. 10.1007/s12178-020-09675-3 PMC7661672 32827301

[18] Y. H. Choi , T. H. Kwon , J. H. Choi , et al., “Factors Associated With Achilles Tendon Re‐Rupture Following Operative Fixation,” Bone & Joint Research 13, no. 7 (2024): 315–320. 10.1302/2046-3758.137.BJR-2023-0258.R1 PMC11214864 38945531

[19] K. Yamaura , Y. Mifune , A. Inui , et al., “Relationship Between Glenohumeral Internal Rotation Deficit and Shoulder Conditions in Professional Baseball Pitchers,” Journal of Shoulder and Elbow Surgery 30, no. 9 (2021): 2073–2081. 10.1016/j.jse.2020.10.041 33290850

[20] C. De Bleecker , S. Vermeulen , C. De Blaiser , et al., “Relationship Between Jump‐Landing Kinematics and Lower Extremity Overuse Injuries in Physically Active Populations: A Systematic Review and Meta‐Analysis,” Sports Medicine 50, no. 8 (2020): 1515–1532. 10.1007/s40279-020-01296-7 32514700

[21] T. Mihata , M. H. McGarry , M. Akeda , et al., “Posterior Shoulder Tightness Can be a Risk Factor of Scapular Malposition: A Cadaveric Biomechanical Study,” Journal of Shoulder and Elbow Surgery 29, no. 1 (2020): 175–184. 10.1016/j.jse.2019.05.040 31420224

[22] C. H. Cho , K. C. Bae , and D. H. Kim , “Incidence and Risk Factors for Early Postoperative Stiffness After Arthroscopic Rotator Cuff Repair in Patients Without Preoperative Stiffness,” Scientific Reports 12, no. 1 (2022): 3132. 10.1038/s41598-022-07123-5 PMC8873420 35210518

[23] P. P. Y. Lui and P. S. H. Yung , “Inflammatory Mechanisms Linking Obesity and Tendinopathy,” Journal of Orthopaedic Translation 31 (2021): 80–90. 10.1016/j.jot.2021.10.003 PMC8666605 34976728

[24] M. S. Kim , G. Y. Jang , and N. S. Cho , “Effect of Preoperative Lipidemic Control on Retear Rates After Rotator Cuff Repair in Patients with Hyperlipidemia,” American Journal of Sports Medicine 52, no. 11 (2024): 2835–2842. 10.1177/03635465241264818 39166295

[25] M. Macchi , M. Spezia , S. Elli , et al., “Obesity Increases the Risk of Tendinopathy, Tendon Tear and Rupture, and Postoperative Complications: A Systematic Review of Clinical Studies,” Clinical Orthopaedics and Related Research 478, no. 8 (2020): 1839–1847. 10.1097/CORR.0000000000001261 PMC7371074 32732565

[26] T. A. Ranger , A. M. Wong , J. L. Cook , et al., “Is There an Association Between Tendinopathy and Diabetes Mellitus? A Systematic Review With Meta‐Analysis,” British Journal of Sports Medicine 50, no. 16 (2016): 982–989. 10.1136/bjsports-2015-094735 26598716

[27] H. T. Leong , S. C. Fu , X. He , et al., “Risk Factors for Rotator Cuff Tendinopathy: A Systematic Review and Meta‐Analysis,” Journal of Rehabilitation Medicine 51, no. 9 (2019): 627–637. 10.2340/16501977-2598 31489438

[28] M. Posthumus , A. V. September , D. O'Cuinneagain , et al., “The COL5A1 Gene Is Associated With Increased Risk of Anterior Cruciate Ligament Ruptures in Female Participants,” American Journal of Sports Medicine 37, no. 11 (2009): 2234–2240. 10.1177/0363546509338266 19654427

[29] G. Nie , X. Wen , X. Liang , et al., “Additional Evidence Supports Association of Common Genetic Variants in MMP3 and TIMP2 With Increased Risk of Chronic Achilles Tendinopathy Susceptibility,” Journal of Science and Medicine in Sport 22, no. 10 (2019): 1074–1078. 10.1016/j.jsams.2019.05.021 31208828

[30] M. V. Sankova , N. M. Beeraka , M. V. Oganesyan , et al., “Recent Developments in Achilles Tendon Risk‐analyzing Rupture Factors for Enhanced Injury Prevention and Clinical Guidance: Current Implications of Regenerative Medicine,” Journal of Orthopaedic Translation 49 (2024): 289–307. 10.1016/j.jot.2024.08.024 PMC11570240 39559294

[31] X. Kang , B. Tian , L. Zhang , et al., “Relationship of Common Variants in MPP7, TIMP2 and CASP8 Genes With the Risk of Chronic Achilles Tendinopathy,” Scientific Reports 9, no. 1 (2019): 17627. 10.1038/s41598-019-54097-y PMC6879592 31772230

[32] L. El Khoury , M. Posthumus , M. Collins , et al., “Polymorphic Variation Within the ADAMTS2, ADAMTS14, ADAMTS5, ADAM12 and TIMP2 Genes and the Risk of Achilles Tendon Pathology: A Genetic Association Study,” Journal of Science and Medicine in Sport 16, no. 6 (2013): 493–498. 10.1016/j.jsams.2013.02.006 23491141

[33] R. Kluger , K. R. Huber , P. G. Seely , et al., “Novel Tenascin‐C Haplotype Modifies the Risk for a Failure to Heal after Rotator Cuff Repair,” American Journal of Sports Medicine 45, no. 13 (2017): 2955–2964. 10.1177/0363546517729810 28952802

[34] L. Y. El Khoury , R. Rickaby , and T. Samiric , “Promoter Methylation Status of the TIMP2 and ADAMTS4 Genes and Patellar Tendinopathy,” Journal of Science and Medicine in Sport 21, no. 4 (2018): 378–382. 10.1016/j.jsams.2017.08.016 28888475

[35] N. L. Millar , D. S. Gilchrist , M. Akbar , et al., “MicroRNA29a Regulates IL‐33‐Mediated Tissue Remodelling in Tendon Disease,” Nature Communications 6 (2015): 6774. 10.1038/ncomms7774 PMC4403384 25857925

[36] K. Willard , S. Mannion , C. J. Saunders , et al., “The Interaction of Polymorphisms in Extracellular Matrix Genes and Underlying miRNA Motifs That Modulate Susceptibility to Anterior Cruciate Ligament Rupture,” Journal of Science and Medicine in Sport 21, no. 1 (2018): 22–28. 10.1016/j.jsams.2017.08.017 28927971

[37] T. Heir and G. Eide , “Injury Proneness in Infantry Conscripts Undergoing a Physical Training Programme: Smokeless Tobacco Use, Higher Age, and Low Levels of Physical Fitness Are Risk Factors,” Scandinavian Journal of Medicine & Science in Sports 7, no. 5 (1997): 304–311. 10.1111/j.1600-0838.1997.tb00158.x 9338950

[38] S. Wahood , O. Alani , I. Draw , et al., “Fluoroquinolones for Dermatologists: A Practical Guide to Clinical Use and Risk Management,” Pharmaceuticals (Basel) 18, no. 6 (2025): 800. 10.3390/ph18060800 PMC12196204 40573197

[39] S. A. Taylor , R. M. Degen , A. E. White , et al., “Risk Factors for Revision Surgery after Superior Labral Anterior‐Posterior Repair: A National Perspective,” American Journal of Sports Medicine 45, no. 7 (2017): 1640–1644. 10.1177/0363546517691950 28282498

[40] J. Zabrzyński , G. Huri , M. Gagat , et al., “The Impact of Smoking on Clinical Results Following the Rotator Cuff and Biceps Tendon Complex Arthroscopic Surgery,” Journal of Clinical Medicine 10, no. 4 (2021): 599. 10.3390/jcm10040599 PMC7915659 33562734

[41] M. R. Bidell and T. P. Lodise , “Fluoroquinolone‐Associated Tendinopathy: Does Levofloxacin Pose the Greatest Risk?,” Pharmacotherapy 36, no. 6 (2016): 679–693. 10.1002/phar.1761 27138564

[42] A. L. Stephenson , W. Wu , D. Cortes , et al., “Tendon Injury and Fluoroquinolone Use: A Systematic Review,” Drug Safety 36, no. 9 (2013): 709–721. 10.1007/s40264-013-0089-8 23888427

[43] C. Alves , D. Mendes , and F. B. Marques , “Fluoroquinolones and the Risk of Tendon Injury: A Systematic Review and Meta‐Analysis,” European Journal of Clinical Pharmacology 75, no. 10 (2019): 1431–1443. 10.1007/s00228-019-02713-1 31270563

[44] F. García‐Heras , J. Rodríguez‐Medina , A. Castañeda , et al., “Occupational Injuries of Spanish Wildland Firefighters: A Descriptive Analysis,” Healthcare (Basel) 12, no. 16 (2024): 1615. 10.3390/healthcare12161615 PMC11354085 39201173

[45] L. Autret , É. Shipley , B. Lodde , et al., “Rheumatic and Musculoskeletal Disorders in Musicians: Risks, Adaptations and Management,” RMD Open 11, no. 4 (2025): e006149. 10.1136/rmdopen-2025-006149 PMC12519710 41083266

[46] C. Zhang , Z. Jia , J. Li , et al., “Impact of Lifestyle and Clinical Factors on the Prognosis of Tennis Elbow,” Scientific Reports 14, no. 1 (2024): 3063. 10.1038/s41598-024-53669-x PMC10847088 38321129

[47] E. Gracey , A. Burssens , I. Cambré , et al., “Tendon and Ligament Mechanical Loading in the Pathogenesis of Inflammatory Arthritis,” Nature Reviews Rheumatology 16, no. 4 (2020): 193–207. 10.1038/s41584-019-0364-x PMC7815340 32080619

[48] R. Gehwolf , H. Tempfer , N. P. Cesur , et al., “Tendinopathy: The Interplay Between Mechanical Stress, Inflammation, and Vascularity,” Advanced Science 12, no. 36 (2025): e06440. 10.1002/advs.202506440 PMC12463082 40855996

[49] A. Peñin‐Franch , J. A. García‐Vidal , C. M. Martínez , et al., “Galvanic Current Activates the NLRP3 Inflammasome to Promote Type I Collagen Production in Tendon,” eLife 11 (2022): e73675. 10.7554/eLife.73675 PMC8896827 35199642

[50] A. Fung , M. Sun , L. J. Soslowsky , et al., “Targeted Conditional Collagen XII Deletion Alters Tendon Function,” Matrix Biology Plus 16 (2022): 100123. 10.1016/j.mbplus.2022.100123 PMC9597098 36311462

[51] Y. Izu , S. M. Adams , B. K. Connizzo , et al., “Collagen XII Mediated Cellular and Extracellular Mechanisms Regulate Establishment of Tendon Structure and Function,” Matrix Biology 95 (2021): 52–67. 10.1016/j.matbio.2020.10.004 PMC7870578 33096204

[52] A. Vinhas , A. F. Almeida , M. T. Rodrigues , et al., “Prospects of Magnetically Based Approaches Addressing Inflammation in Tendon Tissues,” Advanced Drug Delivery Reviews 196 (2023): 114815. 10.1016/j.addr.2023.114815 37001644

[53] S. Chen , Y. Lin , H. Yang , et al., “A CD26+ Tendon Stem Progenitor Cell Population Contributes to Tendon Repair and Heterotopic Ossification,” Nature Communications 16, no. 1 (2025): 749. 10.1038/s41467-025-56112-5 PMC11739514 39820504

[54] M. Zhang , H. Liu , Q. Cui , et al., “Tendon Stem Cell‐derived Exosomes Regulate Inflammation and Promote the High‐quality Healing of Injured Tendon,” Stem Cell Research & Therapy 11, no. 1 (2020): 402. 10.1186/s13287-020-01918-x PMC7499865 32943109

[55] K. Song , T. Jiang , P. Pan , et al., “Exosomes From Tendon Derived Stem Cells Promote Tendon Repair Through miR‐144‐3p‐Regulated Tenocyte Proliferation and Migration,” Stem Cell Research & Therapy 13, no. 1 (2022): 80. 10.1186/s13287-022-02723-4 PMC8867681 35197108

[56] T. Wang , A. Wagner , R. Gehwolf , et al., “Load‐Induced Regulation of Tendon Homeostasis by SPARC, a Genetic Predisposition Factor for Tendon and Ligament Injuries,” Science Translational Medicine 13, no. 582 (2021): eabe5738. 10.1126/scitranslmed.abe5738 33627488

[57] D. Li , S. Li , S. He , et al., “Restoring Tendon Microenvironment in Tendinopathy: Macrophage Modulation and Tendon Regeneration With Injectable Tendon Hydrogel and Tendon‐Derived Stem Cells Exosomes,” Bioactive Materials 47 (2025): 152–169. 10.1016/j.bioactmat.2025.01.016 PMC11791013 39906648

[58] A. J. De Micheli , J. B. Swanson , N. P. Disser , et al., “Single‐cell Transcriptomic Analysis Identifies Extensive Heterogeneity in the Cellular Composition of Mouse Achilles Tendons,” American Journal of Physiology. Cell Physiology 319, no. 5 (2020): C885–C894. 10.1152/ajpcell.00372.2020 PMC7701267 32877217

[59] M. Stańczak , B. Kacprzak , and P. Gawda , “Tendon Cell Biology: Effect of Mechanical Loading,” Cellular Physiology and Biochemistry 58, no. 6 (2024): 677–701. 10.33594/000000743 39568406

[60] R. Mousavizadeh , P. Hojabrpour , F. Eltit , et al., “β1 integrin, ILK and mTOR Regulate Collagen Synthesis in Mechanically Loaded Tendon Cells,” Scientific Reports 10, no. 1 (2020): 12644. 10.1038/s41598-020-69267-6 PMC7387456 32724089

[61] Y. Fang , D. Zhu , J. Wei , et al., “Collagen Denaturation in Post‐run Achilles Tendons and Achilles Tendinopathy: In Vivo Mechanophysiology and Magnetic Resonance Imaging,” Science Advances 10, no. 40 (2024): eado2015. 10.1126/sciadv.ado2015 PMC11446262 39356750

[62] L. Pringels , J. L. Cook , E. Witvrouw , et al., “Exploring the Role of Intratendinous Pressure in the Pathogenesis of Tendon Pathology: A Narrative Review and Conceptual Framework,” British Journal of Sports Medicine 57, no. 16 (2023): 1042–1048. 10.1136/bjsports-2022-106066 PMC10423488 36323498

[63] J. L. Zitnay , A. H. Lin , and J. A. Weiss , “Tendons Exhibit Greater Resistance to Tissue and Molecular‐Level Damage With Increasing Strain Rate During Cyclic Fatigue,” Acta Biomaterialia 134 (2021): 435–442. 10.1016/j.actbio.2021.07.045 PMC8941880 34314889

[64] D. Nie , Y. Zhou , W. Wang , et al., “Mechanical Overloading Induced‐Activation of mTOR Signaling in Tendon Stem/Progenitor Cells Contributes to Tendinopathy Development,” Frontiers in Cell and Developmental Biology 9 (2021): 687856. 10.3389/fcell.2021.687856 PMC8311934 34322484

[65] N. L. Millar , J. H. Reilly , S. C. Kerr , et al., “Hypoxia: A Critical Regulator of Early Human Tendinopathy,” Annals of the Rheumatic Diseases 71, no. 2 (2012): 302–310. 10.1136/ard.2011.154229 21972243

[66] M. F. R. Merkel , R. B. Svensson , J. R. Jakobsen , et al., “Widespread Vascularization and Correlation of Glycosaminoglycan Accumulation to Tendon Pain in Human Plantar Fascia Tendinopathy,” American Journal of Sports Medicine 52, no. 7 (2024): 1834–1844. 10.1177/03635465241246262 38708721

[67] J. H. Wang and I. Komatsu , “Tendon Stem Cells: Mechanobiology and Development of Tendinopathy,” Advances in Experimental Medicine and Biology 920 (2016): 53–62. 10.1007/978-3-319-33943-6_5 27535248

[68] J. L. Cook , E. Rio , C. R. Purdam , et al., “Revisiting the Continuum Model of Tendon Pathology: What Is Its Merit in Clinical Practice and Research?,” British Journal of Sports Medicine 50, no. 19 (2016): 1187–1191. 10.1136/bjsports-2015-095422 PMC5118437 27127294

[69] P. M. Williamson , B. R. Freedman , N. Kwok , et al., “Tendinopathy and Tendon Material Response to Load: What We Can Learn From Small Animal Studies,” Acta Biomaterialia 134 (2021): 43–56. 10.1016/j.actbio.2021.07.046 PMC8542586 34325074

[70] F. S. Passini , P. K. Jaeger , A. S. Saab , et al., “Shear‐Stress Sensing by PIEZO1 Regulates Tendon Stiffness in Rodents and Influences Jumping Performance in Humans,” Nature Biomedical Engineering 5, no. 12 (2021): 1457–1471. 10.1038/s41551-021-00716-x PMC7612848 34031557

[71] D. L. Jones , G. F. Hallström , X. Jiang , et al., “Mechanoepigenetic Regulation of Extracellular Matrix Homeostasis via Yap and Taz,” Proceedings of National Academy of Sciences 120, no. 22 (2023): e2211947120. 10.1073/pnas.2211947120 PMC10235980 37216538

[72] D. Kim , H. Y. Moon , and M. Lee , “Mechanotransduction as a Therapeutic Target in Tendinopathy: Molecular Pathways and Exercise Implications,” Research in Sports Medicine 34, no. 3 (2026): 322–337. 10.1080/15438627.2025.2568936 41046360

[73] A. Savadipour , D. Palmer , E. V. Ely , et al., “The Role of PIEZO Ion Channels in the Musculoskeletal System,” American Journal of Physiology. Cell Physiology 324, no. 3 (2023): C728–C740. 10.1152/ajpcell.00544.2022 PMC10027092 36717101

[74] L. Liu , H. Yu , H. Zhao , et al., “Matrix‐Transmitted Paratensile Signaling Enables Myofibroblast‐Fibroblast Cross Talk in Fibrosis Expansion,” PNAS 117, no. 20 (2020): 10832–10838. 10.1073/pnas.1910650117 PMC7245086 32358190

[75] X. Zhang and C. D. Eliasberg , “Rodeo SA. Mitochondrial Dysfunction and Potential Mitochondrial Protectant Treatments in Tendinopathy,” Annals of the New York Academy of Sciences 1490, no. 1 (2021): 29–41. 10.1111/nyas.14599 33843069

[76] Z. Chen , M. Li , P. Chen , et al., “Mechanical Overload‐Induced Release of Extracellular Mitochondrial Particles From Tendon Cells Leads to Inflammation in Tendinopathy,” Experimental & Molecular Medicine 56, no. 3 (2024): 583–599. 10.1038/s12276-024-01183-5 PMC10985099 38424192

[77] A. C. Abraham , S. A. Shah , M. Golman , et al., “Targeting the NF‐κB Signaling Pathway in Chronic Tendon Disease,” Science Translational Medicine 11, no. 481 (2019): eaav4319. 10.1126/scitranslmed.aav4319 PMC6534967 30814338

[78] G. C. Jones , A. N. Corps , C. J. Pennington , et al., “Expression Profiling of Metalloproteinases and Tissue Inhibitors of Metalloproteinases in Normal and Degenerate Human Achilles Tendon,” Arthritis and Rheumatism 54, no. 3 (2006): 832–842. 10.1002/art.21672 16508964

[79] M. Tsuzaki , G. Guyton , W. Garrett , et al., “IL‐1 Beta Induces COX2, MMP‐1, ‐3 and ‐13, ADAMTS‐4, IL‐1 Beta and IL‐6 in Human Tendon Cells,” Journal of Orthopaedic Research 21, no. 2 (2003): 256–264. 10.1016/S0736-0266(02)00141-9 12568957

[80] Z. Chen , M. Jin , and H. He , “Mesenchymal Stem Cells and Macrophages and Their Interactions in Tendon‐Bone Healing,” Journal of Orthopaedic Translation 39 (2023): 63–73. 10.1016/j.jot.2022.12.005 PMC10175706 37188000

[81] B. Song , C. Jiang , H. Luo , et al., “Macrophage M1 Plays a Positive Role in Aseptic Inflammation‐Related Graft Loosening after Anterior Cruciate Ligament Reconstruction Surgery,” Inflammation 40, no. 6 (2017): 1815–1824. 10.1007/s10753-017-0616-3 28707248

[82] Y. J. Fu , Y. F. Shi , L. Y. Wang , et al., “All‐Natural Immunomodulatory Bioadhesive Hydrogel Promotes Angiogenesis and Diabetic Wound Healing by Regulating Macrophage Heterogeneity,” Advanced Science 10, no. 13 (2023): e2206771. 10.1002/advs.202206771 PMC10161050 36862027

[83] L. Dong , L. Li , Y. Song , et al., “MSC‐Derived Immunomodulatory Extracellular Matrix Functionalized Electrospun Fibers for Mitigating Foreign‐Body Reaction and Tendon Adhesion,” Acta Biomaterialia 133 (2021): 280–296. 10.1016/j.actbio.2021.04.035 33894349

[84] C. N. Manning , C. Martel , S. E. Sakiyama‐Elbert , et al., “Adipose‐Derived Mesenchymal Stromal Cells Modulate Tendon Fibroblast Responses to Macrophage‐Induced Inflammation in Vitro,” Stem Cell Research & Therapy 6, no. 1 (2015): 74. 10.1186/s13287-015-0059-4 PMC4416344 25889287

[85] Y. Zhang , J. Wang , F. Tang , et al., “Mechanistic Insights Into IL‐1β‐Mediated Progression of Tendinopathy,” Frontiers in Immunology 16 (2025): 1657285. 10.3389/fimmu.2025.1657285 PMC12615462 41246304

[86] Y. Zhou , W. Zhang , J. Lin , et al., “Mechanical Stretch Promotes the Neutrophil Recruitment Potential of Fibroblasts Through the Piezo/NFAT1/LIF Axis,” Cell Signalling 131 (2025): 111718. 10.1016/j.cellsig.2025.111718 40086612

[87] M. H. Hoffmann , H. Kirchner , G. Krönke , et al., “Inflammatory Tissue Priming: Novel Insights and Therapeutic Opportunities for Inflammatory Rheumatic Diseases,” Annals of the Rheumatic Diseases 83, no. 10 (2024): 1233–1253. 10.1136/ard-2023-224092 38702177

[88] Y. Zhang , J. Wang , F. Tang , et al., “Mechanistic Insights Into IL‐1β‐mediated Progression of Tendinopathy,” Frontiers in Immunology 16 (2025): 1657285. 10.3389/fimmu.2025.1657285 PMC12615462 41246304

[89] A. Porter , L. Wang , L. Han , et al., “Bio‐Orthogonal Click Chemistry Methods to Evaluate the Metabolism of Inflammatory Challenged Cartilage After Traumatic Overloading,” ACS Biomaterials Science & Engineering 8, no. 6 (2022): 2564–2573. 10.1021/acsbiomaterials.2c00024 PMC10461521 35561285

[90] J. Shen , Y. Chen , Y. Zhang , C. Zhang , and H. Liu , “Multifaceted Roles of IL‐17 in Bone and Tendon Health,” International Journal of Biological Macromolecules 294 (2025): 139498. 10.1016/j.ijbiomac.2025.139498 39756745

[91] X. Mu , R. Gu , M. Tang , et al., “IL‐17 in Wound Repair: Bridging Acute and Chronic Responses,” Cell Communication and Signaling 22, no. 1 (2024): 288. 10.1186/s12964-024-01668-w PMC11129447 38802947

[92] K. Slowikowski , H. N. Nguyen , E. H. Noss , et al., “CUX1 and IκBζ (NFKBIZ) Mediate the Synergistic Inflammatory Response to TNF and IL‐17A in Stromal Fibroblasts,” PNAS 117, no. 10 (2020): 5532–5541. 10.1073/pnas.1912702117 PMC7071902 32079724

[93] J. P. Sherlock , B. Joyce‐Shaikh , S. P. Turner , et al., “IL‐23 Induces Spondyloarthropathy by Acting on ROR‐γt+ CD3+CD4‐CD8‐ Entheseal Resident T Cells,” Nature Medicine 18, no. 7 (2012): 1069–1076. 10.1038/nm.2817 22772566

[94] H. Li , Y. Li , S. Luo , et al., “The Roles and Mechanisms of the NF‐κB Signaling Pathway in Tendon Disorders,” Frontiers in Veterinary Science 11 (2024): 1382239. 10.3389/fvets.2024.1382239 PMC11228182 38978635

[95] J. McHugh , “Targeting NF‐κB in Tendinopathy,” Nature Reviews Rheumatology 15, no. 5 (2019): 251. 10.1038/s41584-019-0206-x 30914773

[96] X. Lin , X. Tian , H. Jiang , et al., “Carpaine Alleviates Tendinopathy in Mice by Promoting the Ubiquitin‐proteasomal Degradation of p65 via Targeting the E3 Ubiquitin Ligase LRSAM1,” Phytomedicine 124 (2024): 155323. 10.1016/j.phymed.2023.155323 38194842

[97] N. L. Millar and G. A. Murrell , “McInnes IB. Inflammatory Mechanisms in Tendinopathy—Towards Translation,” Nature Reviews Rheumatology 13, no. 2 (2017): 110–122. 10.1038/nrrheum.2016.213 28119539

[98] X. Jiao , Y. Zhang , W. Li , et al., “HIF‐1α Inhibition Attenuates Severity of Achilles Tendinopathy by Blocking NF‐κB and MAPK Pathways,” International Immunopharmacology 106 (2022): 108543. 10.1016/j.intimp.2022.108543 35131569

[99] M. Chen , F. Zou , P. Wang , et al., “Dual‐Barb Microneedle With JAK/STAT Inhibitor‐Loaded Nanovesicles Encapsulation for Tendinopathy,” Advanced Healthcare Materials 13, no. 28 (2024): e2401512. 10.1002/adhm.202401512 39030889

[100] G. Moschini , A. G. Mohanan , I. S. Niewczas , et al., “HIF1α Gates Tendon Response to Overload and Drives Tendinopathy Independently of Vascular Recruitment,” Science Translational Medicine 18, no. 831 (2026): eadt1228. 10.1126/scitranslmed.adt1228 41499520

[101] S. Vasta , A. Di Martino , B. Zampogna , et al., “Role of VEGF, Nitric Oxide, and Sympathetic Neurotransmitters in the Pathogenesis of Tendinopathy: A Review of the Current Evidences,” Frontiers in Aging Neuroscience 8 (2016): 186. 10.3389/fnagi.2016.00186 PMC4977280 27555817

[102] S. Jeon , S. Cho , S. Yoo , et al., “Controlled Delivery of HIF‐1α via Extracellular Vesicles With Collagen‐Binding Activity for Enhanced Wound Healing,” Journal of Controlled Release 380 (2025): 330–347. 10.1016/j.jconrel.2025.02.010 39921033

[103] S. Y. Oh , D. K. Kim , S. H. Han , et al., “Sustained Exposure of Substance P Causes Tendinopathy,” International Journal of Molecular Sciences 21, no. 22 (2020): 8633. 10.3390/ijms21228633 PMC7709031 33207770

[104] N. Schizas , Ø. Lian , F. Frihagen , et al., “Coexistence of Up‐regulated NMDA Receptor 1 and Glutamate on Nerves, Vessels and Transformed Tenocytes in Tendinopathy,” Scandinavian Journal of Medicine & Science in Sports 20, no. 2 (2010): 208–215. 10.1111/j.1600-0838.2009.00913.x 19422642

[105] S. Y. Oh , D. K. Kim , S. H. Han , et al., “Sustained Exposure of Substance P Causes Tendinopathy,” International Journal of Molecular Sciences 21, no. 22 (2020): 8633. 10.3390/ijms21228633 PMC7709031 33207770

[106] V. G. Festinese , M. Faydaver , D. Nardinocchi , et al., “Neural Markers Predict Tendon Healing Outcomes in an Ovine Achilles Tendon Injury Model: Spontaneous Repair versus Amniotic Epithelial Cell‐Induced Regeneration,” International Journal of Molecular Sciences 26, no. 6 (2025): 2445. 10.3390/ijms26062445 PMC11942428 40141090

[107] P. M. Muljadi and N. Andarawis‐Puri , “Glycosaminoglycans Modulate Microscale Mechanics and Viscoelasticity in Fatigue Injured Tendons,” Journal of Biomechanics 152 (2023): 111584. 10.1016/j.jbiomech.2023.111584 37080079

[108] R. Yamashita , S. Tsutsui , S. Mizumoto , et al., “CANT1 Is Involved in Collagen Fibrogenesis in Tendons by Regulating the Synthesis of Dermatan/Chondroitin Sulfate Attached to the Decorin Core Protein,” International Journal of Molecular Sciences 26, no. 6 (2025): 2463. 10.3390/ijms26062463 PMC11941851 40141107

[109] M. Kjaer , “Role of Extracellular Matrix in Adaptation of Tendon and Skeletal Muscle to Mechanical Loading,” Physiological Reviews 84, no. 2 (2004): 649–698. 10.1152/physrev.00031.2003 15044685

[110] D. Chen , Y. Du , J. Llewellyn , et al., “Versican Binds Collagen via Its G3 Domain and Regulates the Organization and Mechanics of Collagenous Matrices,” Journal of Biological Chemistry 300, no. 12 (2024): 107968. 10.1016/j.jbc.2024.107968 PMC11626796 39510178

[111] Y. Cho , H. S. Kim , D. Kang , et al., “CTRP3 Exacerbates Tendinopathy by Dysregulating Tendon Stem Cell Differentiation and Altering Extracellular Matrix Composition,” Science Advances 7, no. 47 (2021): eabg6069. 10.1126/sciadv.abg6069 PMC8604415 34797714

[112] M. Chatterjee , P. M. Muljadi , and N. Andarawis‐Puri , “The Role of the Tendon ECM in Mechanotransduction: Disruption and Repair Following Overuse,” Connective Tissue Research 63, no. 1 (2022): 28–42. 10.1080/03008207.2021.1925663 34030531

[113] K. M. Doersch , D. Barnett , A. Chase , et al., “The Contribution of the Immune System to Genitourinary Fibrosis,” Experimental Biology and Medicine 247, no. 9 (2022): 765–778. 10.1177/15353702221090872 PMC9134766 35531654

[114] J. Deng , S. J. Breda , D. Eygendaal , et al., “Association between Physical Tests and Patients‐Reported Outcomes in Athletes Performing Exercise Therapy for Patellar Tendinopathy: A Secondary Analysis of the JUMPER Study,” American Journal of Sports Medicine 51, no. 13 (2023): 3523–3532. 10.1177/03635465231200241 PMC10623612 37815096

[115] T. S. O. Sleeswijk Visser , E. M. van Es , D. E. Meuffels , et al., “Standardized Pain Mapping for Diagnosing Achilles Tendinopathy,” Journal of Science and Medicine in Sport 25, no. 3 (2022): 204–208. 10.1016/j.jsams.2021.10.010 34753666

[116] M. Bakkegaard , F. E. Johannsen , B. Højgaard , et al., “Ultrasonography as a Prognostic and Objective Parameter in Achilles Tendinopathy: A Prospective Observational Study,” European Journal of Radiology 84, no. 3 (2015): 458–462. 10.1016/j.ejrad.2014.11.028 25577603

[117] R. L. Chimenti , P. C. Chimenti , M. R. Buckley , et al., “Utility of Ultrasound for Imaging Osteophytes in Patients with Insertional Achilles Tendinopathy,” Archives of Physical Medicine and Rehabilitation 97, no. 7 (2016): 1206–1209. 10.1016/j.apmr.2015.12.009 PMC4921278 26740063

[118] D. Ruffino , M. Alfonso , V. Campana , et al., “Sensitivity to Change and Responsiveness of Provocative Load Tests Among Athletes With Patellar Tendinopathy,” Brazilian Journal of Physical Therapy 28, no. 2 (2024): 101064. 10.1016/j.bjpt.2024.101064 PMC11070825 38696973

[119] W. Matthews , R. Ellis , J. Furness , et al., “The Clinical Diagnosis of Achilles Tendinopathy: A Scoping Review,” PeerJ 9 (2021): e12166. 10.7717/peerj.12166 PMC8485842 34692248

[120] K. G. Silbernagel , R. Thomeé , B. I. Eriksson , et al., “Continued Sports Activity, Using a Pain‐Monitoring Model, During Rehabilitation in Patients With Achilles Tendinopathy: A Randomized Controlled Study,” American Journal of Sports Medicine 35, no. 6 (2007): 897–906. 10.1177/0363546506298279 17307888

[121] H. Lohrer and T. Nauck , “Cross‐Cultural Adaptation and Validation of the VISA‐A Questionnaire for German‐Speaking Achilles Tendinopathy Patients,” BMC Musculoskeletal Disorders 10 (2009): 134. 10.1186/1471-2474-10-134 PMC2776582 19878572

[122] M. Bahari , M. Hadadi , A. R. Vosoughi , et al., “Cross‐Cultural Adaptation, Reliability and Validity of the Persian Version of the Victorian Institute of Sport Assessment‐Achilles Questionnaire (VISA‐A),” Disability and Rehabilitation 44, no. 6 (2022): 983–991. 10.1080/09638288.2020.1781268 32664757

[123] M. C. Murphy , F. McCleary , D. Hince , et al., “TENDINopathy Severity Assessment‐Achilles (TENDINS‐A): Evaluation of Reliability and Validity in Accordance With COSMIN Recommendations,” British Journal of Sports Medicine 58, no. 12 (2024): 665–673. 10.1136/bjsports-2023-107741 38575200

[124] C. Stubbs , S. Mc Auliffe , A. Mallows , et al., “The Strength of Association Between Psychological Factors and Clinical Outcome in Tendinopathy: A Systematic Review,” PLoS One 15, no. 11 (2020): e0242568. 10.1371/journal.pone.0242568 PMC7703922 33253233

[125] M. L. Plinsinga , B. K. Coombes , R. Mellor , et al., “Psychological Factors Not Strength Deficits Are Associated With Severity of Gluteal Tendinopathy: A Cross‐Sectional Study,” European Journal of Pain 22, no. 6 (2018): 1124–1133. 10.1002/ejp.1199 29427310

[126] H. B. Sigurðsson and K. Grävare Silbernagel , “Is the VISA‐A Still Seaworthy, or Is It in Need of Maintenance?,” Orthopaedic Journal of Sports Medicine 10, no. 8 (2022): 23259671221108950. 10.1177/23259671221108950 PMC9380230 35982828

[127] M. Gatz , D. Bode , M. Betsch , et al., “Multimodal Ultrasound versus MRI for the Diagnosis and Monitoring of Achilles Tendinopathy: A Prospective Longitudinal Study,” Orthopaedic Journal of Sports Medicine 9, no. 4 (2021): 23259671211006826. 10.1177/23259671211006826 PMC8047827 33912619

[128] F. Mersmann , N. Pentidis , M. S. Tsai , et al., “Patellar Tendon Strain Associates to Tendon Structural Abnormalities in Adolescent Athletes,” Frontiers in Physiology 10 (2019): 963. 10.3389/fphys.2019.00963 PMC6687848 31427983

[129] L. Risch , J. Stoll , A. Schomöller , et al., “Intraindividual Doppler Flow Response to Exercise Differs between Symptomatic and Asymptomatic Achilles Tendons,” Frontiers in Physiology 12 (2021): 617497. 10.3389/fphys.2021.617497 PMC8290144 34295255

[130] L. Risch , M. Cassel , and F. Mayer , “Acute Effect of Running Exercise on Physiological Achilles Tendon Blood Flow,” Scandinavian Journal of Medicine & Science in Sports 28, no. 1 (2018): 138–143. 10.1111/sms.12874 28294413

[131] L. Risch , F. Mayer , and M. Cassel , “Doppler Flow Response Following Running Exercise Differs between Healthy and Tendinopathic Achilles Tendons,” Frontiers in Physiology 12 (2021): 650507. 10.3389/fphys.2021.650507 PMC8021791 33833692

[132] A. Carrozzo , J. Shatrov , and A. El Helou , “Ultrasound‐guided Electrocoagulation of Neovascularisation for Persistent Patellar Tendinopathy in Athletes: A Cohort Study of 25 Patients With a Mean Follow‐up of 5 Years From the SANTI Study Group,” BMJ Open Sport & Exercise Medicine 10, no. 1 (2024): e001900. 10.1136/bmjsem-2024-001900 PMC10946354 38500579

[133] F. Johannsen , S. Jensen , and E. Wetke , “10‐year Follow‐up After Standardised Treatment for Achilles Tendinopathy,” BMJ Open Sport & Exercise Medicine 4, no. 1 (2018): e000415. 10.1136/bmjsem-2018-000415 PMC6173232 30305926

[134] M. Rehman , F. Duarte Silva , and A. Chhabra , “Diagnostic Efficacy of Posterior Tibialis Tendon Dysfunction: A Systematic Review of Literature,” European Radiology 34, no. 5 (2024): 3513–3523. 10.1007/s00330-023-10364-1 37889271

[135] Y. Zhu , B. Dai , S. Zhang , et al., “Tissue Mimetic Membranes for Healing Augmentation of Tendon‐Bone Interface in Rotator Cuff Repair,” Advanced Materials 37, no. 10 (2025): e2407358. 10.1002/adma.202407358 PMC11899491 39888084

[136] F. G. Thankam , Z. K. Roesch , M. F. Dilisio , et al., “Association of Inflammatory Responses and ECM Disorganization With HMGB1 Upregulation and NLRP3 Inflammasome Activation in the Injured Rotator Cuff Tendon,” Scientific Reports 8, no. 1 (2018): 8918. 10.1038/s41598-018-27250-2 PMC5995925 29891998

[137] S. Liu , Y. Xie , Q. Chen , et al., “Tendon Healing Progression Evaluated with Magnetic Resonance Imaging Signal Intensity and Its Correlation with Clinical Outcomes within 1 Year after Rotator Cuff Repair with the Suture‐Bridge Technique,” American Journal of Sports Medicine 48, no. 3 (2020): 697–705. 10.1177/0363546519899357 32023083

[138] D. J. Lin , C. J. Burke , B. Abiri , et al., “Supraspinatus Muscle Shear Wave Elastography (SWE): Detection of Biomechanical Differences With Varying Tendon Quality Prior to Gray‐scale Morphologic Changes,” Skeletal Radiology 49, no. 5 (2020): 731–738. 10.1007/s00256-019-03334-6 31811348

[139] H. Uehara , Y. Itoigawa , T. Wada , et al., “Shear Wave Elastography Correlates to Degeneration and Stiffness of the Long Head of the Biceps Tendon in Patients Undergoing Tenodesis With Arthroscopic Shoulder Surgery,” Journal of Shoulder and Elbow Surgery 33, no. 1 (2024): e31–e41. 10.1016/j.jse.2023.05.014 37327988

[140] B. Frankewycz , L. Henssler , J. Weber , et al., “Changes of Material Elastic Properties During Healing of Ruptured Achilles Tendons Measured With Shear Wave Elastography: A Pilot Study,” International Journal of Molecular Sciences 21, no. 10 (2020): 3427. 10.3390/ijms21103427 PMC7279368 32408704

[141] Q. Steiner , A. Wang , L. Slane , et al., “Ultrasound Quantitative Characterization of Tendinopathy With Shear Wave Elastography in an Ex Vivo Porcine Tendon Model,” European Radiology Experimental 9, no. 1 (2025): 33. 10.1186/s41747-024-00542-1 PMC11926283 40111644

[142] W. A. Berrigan , K. Cipriano , K. A. Easley , et al., “Quantifying Mechanical Properties of the Patellar and Achilles Tendons Using Ultrasound Shear Wave Elastography: A Pilot Study,” Diagnostics (Basel) 15, no. 7 (2025): 879. 10.3390/diagnostics15070879 PMC11988425 40218229

[143] L. N. Zhang , W. B. Wan , Y. X. Wang , et al., “Evaluation of Elastic Stiffness in Healing Achilles Tendon after Surgical Repair of a Tendon Rupture Using in Vivo Ultrasound Shear Wave Elastography,” Medical Science Monitor 22 (2016): 1186–1191. 10.12659/MSM.895674 PMC4835154 27072885

[144] W. Yang , Q. Yu , Y. Wang , et al., “The Clinical Value of Real‐Time Shear Wave Elastography in Evaluating Closed Complete Achilles Tendon Ruptures,” Journal of Clinical Ultrasound 53, no. 9 (2025): 2100–2107. 10.1002/jcu.70010 40693642

[145] C. C. Ooi , S. K. Wong , V. C. Ma , et al., “The Prevalence of Neovascularity in Rotator Cuff Tendinopathy: Comparing Conventional Doppler With Superb Microvascular Imaging,” Clinical Radiology 77, no. 6 (2022): e442–e448. 10.1016/j.crad.2022.03.003 35428470

[146] S. Takeuchi , B. B. Rothrauff , R. Kanto , et al., “Superb Microvascular Imaging (SMI) Detects Increased Vascularity of the Torn Anterior Cruciate Ligament,” Knee Surgery, Sports Traumatology, Arthroscopy 30, no. 1 (2022): 93–101. 10.1007/s00167-021-06640-6 34121144

[147] S. G. Dakin , J. Newton , F. O. Martinez , et al., “Chronic Inflammation Is a Feature of Achilles Tendinopathy and Rupture,” British Journal of Sports Medicine 52, no. 6 (2018): 359–367. 10.1136/bjsports-2017-098161 PMC5867427 29118051

[148] F. Klatte‐Schulz , S. Minkwitz , A. Schmock , et al., “Different Achilles Tendon Pathologies Show Distinct Histological and Molecular Characteristics,” International Journal of Molecular Sciences 19, no. 2 (2018): 404. 10.3390/ijms19020404 PMC5855626 29385715

[149] C. Darrieutort‐Laffite , F. Blanchard , L. J. Soslowsky , et al., “Biology and Physiology of Tendon Healing,” Joint, Bone, Spine 91, no. 5 (2024): 105696. 10.1016/j.jbspin.2024.105696 38307405

[150] B. Wang , Z. Li , R. Zhou , et al., “Causal Relationship Between Interleukin‐6 Levels and the Risk of Synovitis and Tenosynovitis: A Two‐sample Mendelian Randomization Study,” Medicine (Baltimore) 104, no. 41 (2025): e44811. 10.1097/MD.0000000000044811 PMC12517909 41088586

[151] V. K. Morya , H. W. Lee , C. W. Park , et al., “Computational Analysis of miR‐140 and miR‐135 as Potential Targets to Develop Combinatorial Therapeutics for Degenerative Tendinopathy,” Clinics in Orthopedic Surgery 15, no. 3 (2023): 463–476. 10.4055/cios22237 PMC10232305 37274502

[152] L. Ding , M. Wang , S. Qin , et al., “The Roles of MicroRNAs in Tendon Healing and Regeneration,” Frontiers in Cell and Developmental Biology 9 (2021): 687117. 10.3389/fcell.2021.687117 PMC8283311 34277629

[153] E. K. Rio , C. L. Rabusin , S. E. Munteanu , et al., “Where is Your Pain? Achilles Tendinopathy Pain Location on Loading Is Different to Palpation, Imaging and Recall Location,” Journal of Orthopaedic and Sports Physical Therapy 54, no. 1 (2024): 86–94. 10.2519/jospt.2023.12131 38060345

[154] P. Malliaras , K. Gravare Silbernagel , R. J. de Vos , et al., “Diagnostic Domains, Differential Diagnosis and Conditions Requiring Further Medical Attention That Are Considered Important in the Assessment for Achilles Tendinopathy: A Delphi Consensus Study,” British Journal of Sports Medicine 59, no. 13 (2025): 891–901. 10.1136/bjsports-2024-109185 40240126

[155] D. Skovgaard , V. D. Siersma , S. B. Klausen , et al., “Chronic Hyperglycemia, Hypercholesterolemia, and Metabolic Syndrome Are Associated With Risk of Tendon Injury,” Scandinavian Journal of Medicine & Science in Sports 31, no. 9 (2021): 1822–1831. 10.1111/sms.13984 33963621

[156] R. L. Chimenti , A. A. Post , E. K. Rio , et al., “The Effects of Pain Science Education plus Exercise on Pain and Function in Chronic Achilles Tendinopathy: A Blinded, Placebo‐Controlled, Explanatory, Randomized Trial,” Pain 164, no. 1 (2023): e47–e65. 10.1097/j.pain.0000000000002720 PMC10016230 36095045

[157] L. Pringels , R. Capelleman , A. Van den Abeele , et al., “Effectiveness of Reducing Tendon Compression in the Rehabilitation of Insertional Achilles Tendinopathy: A Randomised Clinical Trial,” British Journal of Sports Medicine 59, no. 9 (2025): 640–650. 10.1136/bjsports-2024-109138 PMC12171478 40011018

[158] A. Funaro , V. Shim , I. Mylle , et al., “Subject‐Specific Biomechanics Influences Tendon Strains in Patients With Achilles Tendinopathy,” Scientific Reports 15, no. 1 (2025): 1084. 10.1038/s41598-024-84202-9 PMC11707288 39774794

[159] C. H. Yeh , J. D. Calder , J. Antflick , et al., “Maximum Dorsiflexion Increases Achilles Tendon Force During Exercise for Midportion Achilles Tendinopathy,” Scandinavian Journal of Medicine & Science in Sports 31, no. 8 (2021): 1674–1682. 10.1111/sms.13974 33864294

[160] P. Malliaras , “Physiotherapy Management of Achilles Tendinopathy,” Journal of Physiotherapy 68, no. 4 (2022): 221–237. 10.1016/j.jphys.2022.09.010 36274038

[161] D. Plotkin , M. Coleman , D. Van Every , et al., “Progressive overload without progressing load? The Effects of Load or Repetition Progression on Muscular Adaptations,” PeerJ 10 (2022): e14142. 10.7717/peerj.14142 PMC9528903 36199287

[162] Y. Demangeot , S. O'Neill , and F. Degache , “Exercise Parameters to Consider for Achilles Tendinopathy: A Modified Delphi Study With International Experts,” British Journal of Sports Medicine 59, no. 19 (2025): 1337–1349. 10.1136/bjsports-2025-110183 PMC12573378 40877027

[163] K. Merry , M. MacPherson , M. Vis‐Dunbar , et al., “Identifying Characteristics of Resistance‐Based Therapeutic Exercise Interventions for Achilles Tendinopathy: A Scoping Review,” Physical Therapy in Sport 63 (2023): 73–94. 10.1016/j.ptsp.2023.06.002 37536026

[164] K. Merry , C. Napier , C. M. Waugh , et al., “Foundational Principles and Adaptation of the Healthy and Pathological Achilles Tendon in Response to Resistance Exercise: A Narrative Review and Clinical Implications,” Journal of Clinical Medicine 11, no. 16 (2022): 4722. 10.3390/jcm11164722 PMC9410084 36012960

[165] A. Irby , J. Gutierrez , C. Chamberlin , et al., “Clinical Management of Tendinopathy: A Systematic Review of Systematic Reviews Evaluating the Effectiveness of Tendinopathy Treatments,” Scandinavian Journal of Medicine & Science in Sports 30, no. 10 (2020): 1810–1826. 10.1111/sms.13734 32484976

[166] K. Lyu , X. Liu , L. Jiang , et al., “The Functions and Mechanisms of Low‐Level Laser Therapy in Tendon Repair (Review),” Frontiers in Physiology 13 (2022): 808374. 10.3389/fphys.2022.808374 PMC8886125 35242050

[167] V. Korakakis , R. Whiteley , A. Tzavara , et al., “The Effectiveness of Extracorporeal Shockwave Therapy in Common Lower Limb Conditions: A Systematic Review Including Quantification of Patient‐Rated Pain Reduction,” British Journal of Sports Medicine 52, no. 6 (2018): 387–407. 10.1136/bjsports-2016-097347 28954794

[168] F. Rosso , D. E. Bonasia , A. Marmotti , et al., “Mechanical Stimulation (Pulsed Electromagnetic Fields “PEMF” and Extracorporeal Shock Wave Therapy “ESWT”) and Tendon Regeneration: A Possible Alternative,” Frontiers in Aging Neuroscience 7 (2015): 211. 10.3389/fnagi.2015.00211 PMC4637423 26617513

[169] R. Charles , L. Fang , R. Zhu , et al., “The Effectiveness of Shockwave Therapy on Patellar Tendinopathy, Achilles Tendinopathy, and Plantar Fasciitis: A Systematic Review and Meta‐analysis,” Frontiers in Immunology 14 (2023): 1193835. 10.3389/fimmu.2023.1193835 PMC10468604 37662911

[170] Y. Chen , K. Lyu , J. Lu , et al., “Biological Response of Extracorporeal Shock Wave Therapy to Tendinopathy in Vivo (review),” Frontiers in Veterinary Science 9 (2022): 851894. 10.3389/fvets.2022.851894 PMC9356378 35942112

[171] M. Stania , G. Juras , D. Chmielewska , et al., “Extracorporeal Shock Wave Therapy for Achilles Tendinopathy,” BioMed Research International 2019 (2019): 3086910. 10.1155/2019/3086910 PMC6948318 31950037

[172] F. U. Verstraelen , N. J. In den Kleef , L. Jansen , et al., “High‐energy versus Low‐energy Extracorporeal Shock Wave Therapy for Calcifying Tendinitis of the Shoulder: Which Is Superior? A Meta‐Analysis,” Clinical Orthopaedics and Related Research 472, no. 9 (2014): 2816–2825. 10.1007/s11999-014-3680-0 PMC4117900 24872197

[173] Y. Fan , Z. Feng , J. Cao , et al., “Efficacy of Extracorporeal Shock Wave Therapy for Achilles Tendinopathy: A Meta‐Analysis,” Orthopaedic Journal of Sports Medicine 8, no. 2 (2020): 2325967120903430. 10.1177/2325967120903430 PMC7686643 33283015

[174] F. Da Ré Guerra , C. P. Vieira , P. P. Marques , et al., “Low Level Laser Therapy Accelerates the Extracellular Matrix Reorganization of Inflamed Tendon,” Tissue & Cell 49, no. 4 (2017): 483–488. 10.1016/j.tice.2017.05.006 28606650

[175] Z. Huang , J. Ma , J. Chen , et al., “The Effectiveness of Low‐level Laser Therapy for Nonspecific Chronic Low Back Pain: A Systematic Review and Meta‐Analysis,” Arthritis Research & Therapy 17 (2015): 360. 10.1186/s13075-015-0882-0 PMC4704537 26667480

[176] B. Girgis and J. A. Duarte , “Physical Therapy for Tendinopathy: An Umbrella Review of Systematic Reviews and Meta‐Analyses,” Physical Therapy in Sport 46 (2020): 30–46. 10.1016/j.ptsp.2020.08.002 32877858

[177] A. T. Doyle , C. Lauber , and K. Sabine , “The Effects of Low‐Level Laser Therapy on Pain Associated with Tendinopathy: A Critically Appraised Topic,” Journal of Sport Rehabilitation 25, no. 1 (2016): 83–90. 10.1123/jsr.2014-0219 25559198

[178] B. W. D. Yap and E. C. W. Lim , “Shedding More Light on the Short‐Term Effect of Low‐Level Laser Therapy on Pain in Tendinopathy: A Systematic Review With Meta‐analysis,” Journal of Back and Musculoskeletal Rehabilitation 38, no. 6 (2025): 1232–1256. 10.1177/10538127251339104 40437920

[179] S. Rayegani , M. Bahrami , B. Samadi , et al., “Comparison of the Effects of Low Energy Laser and Ultrasound in Treatment of Shoulder Myofascial Pain Syndrome: A Randomized Single‐Blinded Clinical Trial,” European Journal of Physical and Rehabilitation Medicine 47, no. 3 (2011): 381–389. 21946400

[180] P. Ö. Başaran and D. E. Büyükşireci , “Comparison of Low‐level Laser Therapy versus Neuromuscular Electrical Nerve Stimulation at Hemiplegic Shoulder Pain and Upper Extremity Functions,” Lasers in Medical Science 40, no. 1 (2025): 42. 10.1007/s10103-025-04305-1 PMC11761826 39853536

[181] M. H. Kim , W. Martin , A. Quarmby , et al., “Effects of Sensorimotor Training on Functional and Pain Outcomes in Achilles Tendinopathy: A Systematic Review,” Frontiers in Sports and Active Living 6 (2024): 1414633. 10.3389/fspor.2024.1414633 PMC11306088 39119510

[182] H. Lohrer , “Minimum 3.5‐year Outcomes of Operative Treatment for Achilles Tendon Partial Tears in the Midportion and Retrocalcaneal Area,” Journal of Orthopaedic Surgery and Research 15, no. 1 (2020): 395. 10.1186/s13018-020-01856-7 PMC7488251 32912202

[183] G. Nicholson , J. Walker , Z. Dawson , et al., “Morphological and Functional Outcomes of Operatively Treated Achilles Tendon Ruptures,” Physician and Sportsmedicine 48, no. 3 (2020): 290–297. 10.1080/00913847.2019.1685364 31662010

[184] R. M. Biedert and P. M. Tscholl , “Surgical Treatment of Chronic Proximal Patellar Tendon Tears Grades 3 and 4 Using Augmentation With Quadriceps Tendon‐bone Graft,” Knee 45 (2023): 54–64. 10.1016/j.knee.2023.09.004 37806246

[185] D. J. Cognetti , A. J. Sheean , J. W. Arner , et al., “Surgical Management of Patellar Tendinopathy Results in Improved Outcomes and High Rates of Return to Sport: A Systematic Review,” Journal of Knee Surgery 36, no. 11 (2023): 1171–1190. 10.1055/s-0042-1757701 36162426

[186] P. Y. Chen , I. S. Tzeng , K. C. Yang , et al., “Endoscopic versus Open Surgery for Insertional Achilles Tendinopathy: A Systematic Review and Meta‐analysis of Comparative Outcomes,” Journal of Experimental Orthopaedics 12, no. 3 (2025): e70374. 10.1002/jeo2.70374 PMC12287823 40708889

[187] H. Thermann , R. Fischer , N. Gougoulias , et al., “Endoscopic Debridement for Non‐Insertional Achilles Tendinopathy With and Without Platelet‐rich Plasma,” Journal of Sport and Health Science 12, no. 2 (2023): 275–280. 10.1016/j.jshs.2020.06.012 PMC10105028 32619656

[188] T. K. Lee and S. J. Shin , “Functional Recovery of the Shoulder After Arthroscopic Treatment for Chronic Calcific Tendinitis,” Clinics in Shoulder and Elbow 21, no. 2 (2018): 75–81. 10.5397/cise.2018.21.2.75 PMC7726381 33330156

[189] T. Philips , M. Dejonghe , and D. Petre , “Arthroscopic Treatment of Calcific Tendinitis Without Rotator Cuff Repair Using Prospectively Collected Results,” Acta Orthopaedica Belgica 89, no. 2 (2023): 195–200. 10.52628/89.2.9574 37924534

[190] M. Kroslak and G. A. C. Murrell , “Surgical Treatment of Lateral Epicondylitis: A Prospective, Randomized, Double‐Blinded, Placebo‐Controlled Clinical Trial,” American Journal of Sports Medicine 46, no. 5 (2018): 1106–1113. 10.1177/0363546517753385 29498885

[191] S. Ketola , J. T. Lehtinen , and I. Arnala , “Arthroscopic Decompression Not Recommended in the Treatment of Rotator Cuff Tendinopathy: A Final Review of a Randomised Controlled Trial at a Minimum Follow‐Up of Ten Years,” Bone & Joint Journal 99‐B, no. 6 (2017): 799–805. 10.1302/0301-620X.99B6.BJJ-2016-0569.R1 28566400

[192] J. Kirschner and B. Hunter , “In Acute Soft Tissue Injuries, NSAIDs Do Not Differ From Acetaminophen for Pain Reduction,” Annals of Internal Medicine 173, no. 12 (2020): JC66. 10.7326/ACPJ202012150-066 33316182

[193] C. H. Liao , L. P. Lin , T. Y. Yu , et al., “Ibuprofen Inhibited Migration of Skeletal Muscle Cells in Association With Downregulation of p130cas and CrkII Expressions,” Skeletal Muscle 9, no. 1 (2019): 23. 10.1186/s13395-019-0208-z PMC6714350 31464636

[194] Z. J. Foster , T. T. Voss , J. Hatch , et al., “Corticosteroid Injections for Common Musculoskeletal Conditions,” American Family Physician 92, no. 8 (2015): 694–699. 26554409

[195] K. Huang , G. Giddins , and L. D. Wu , “Platelet‐Rich Plasma versus Corticosteroid Injections in the Management of Elbow Epicondylitis and Plantar Fasciitis: An Updated Systematic Review and Meta‐Analysis,” American Journal of Sports Medicine 48, no. 10 (2020): 2572–2585. 10.1177/0363546519888450 31821010

[196] Z. Ye , Y. Yuan , G. Kuang , et al., “Platelet‐Rich Plasma and Corticosteroid Injection for Tendinopathy: A Systematic Review and Meta‐Analysis,” BMC Musculoskeletal Disorders 26, no. 1 (2025): 339. 10.1186/s12891-025-08566-3 PMC11980122 40200209

[197] A. Scott , R. F. LaPrade , K. G. Harmon , et al., “Platelet‐Rich Plasma for Patellar Tendinopathy: A Randomized Controlled Trial of Leukocyte‐Rich PRP or Leukocyte‐Poor PRP versus Saline,” American Journal of Sports Medicine 47, no. 7 (2019): 1654–1661. 10.1177/0363546519837954 31038979

[198] A. Barman , M. K. Sinha , J. Sahoo , et al., “Platelet‐rich Plasma Injection in the Treatment of Patellar Tendinopathy: A Systematic Review and Meta‐analysis,” Knee Surgery & Related Research 34, no. 1 (2022): 22. 10.1186/s43019-022-00151-5 PMC9066802 35509070

[199] E. S. R. Barreto , C. R. Antunes Júnior , and I. C. Silva , “Is Platelet‐rich Plasma Effective in Treating Achilles Tendinopathy? A Meta‐analysis of Randomized Clinical Trials,” Clinical Orthopaedics and Related Research 483, no. 5 (2025): 779–790. 10.1097/CORR.0000000000003349 PMC12014056 39745256

[200] Y. J. Zhang , S. Z. Xu , P. C. Gu , et al., “Is Platelet‐rich Plasma Injection Effective for Chronic Achilles Tendinopathy?,” A Clinical Orthopaedics and Related Research 476, no. 8 (2018): 1633–1641. 10.1007/s11999.0000000000000258 PMC6259774 29601383

[201] G. Jiang , Y. Wu , J. Meng , et al., “Comparison of Leukocyte‐Rich Platelet‐Rich Plasma and Leukocyte‐Poor Platelet‐Rich Plasma on Achilles Tendinopathy at an Early Stage in a Rabbit Model,” American Journal of Sports Medicine 48, no. 5 (2020): 1189–1199. 10.1177/0363546520906142 32134682

[202] Y. Zhou , J. Zhang , H. Wu , et al., “The Differential Effects of Leukocyte‐Containing and Pure Platelet‐Rich Plasma (PRP) on Tendon Stem/Progenitor Cells—Implications of PRP Application for the Clinical Treatment of Tendon Injuries,” Stem Cell Research & Therapy 6, no. 1 (2015): 173. 10.1186/s13287-015-0172-4 PMC4572462 26373929

[203] L. Zhang , S. Chen , P. Chang , et al., “Harmful Effects of Leukocyte‐Rich Platelet‐Rich Plasma on Rabbit Tendon Stem Cells in Vitro,” American Journal of Sports Medicine 44, no. 8 (2016): 1941–1951. 10.1177/0363546516644718 27184544

[204] J. A. Cross , B. J. Cole , K. P. Spatny , et al., “Leukocyte‐Reduced Platelet‐Rich Plasma Normalizes Matrix Metabolism in Torn Human Rotator Cuff Tendons,” American Journal of Sports Medicine 43, no. 12 (2015): 2898–2906. 10.1177/0363546515608157 26460099

[205] A. D. K. Le , L. Enweze , M. R. DeBaun , et al., “Current Clinical Recommendations for Use of Platelet‐Rich Plasma,” Current Reviews in Musculoskeletal Medicine 11, no. 4 (2018): 624–634. 10.1007/s12178-018-9527-7 PMC6220007 30353479

[206] A. P. Boesen , R. Hansen , M. I. Boesen , et al., “Effect of High‐Volume Injection, Platelet‐Rich Plasma, and Sham Treatment in Chronic Midportion Achilles Tendinopathy: A Randomized Double‐Blinded Prospective Study,” American Journal of Sports Medicine 45, no. 9 (2017): 2034–2043. 10.1177/0363546517702862 28530451

[207] R. Hassan , D. Poku , N. Miah , et al., “High‐Volume Injections in Achilles Tendinopathy: A Systematic Review,” British Medical Bulletin 152, no. 1 (2024): 35–47. 10.1093/bmb/ldae015 39496560

[208] A. P. Boesen , H. Langberg , R. Hansen , et al., “High Volume Injection With and Without Corticosteroid in Chronic Midportion Achilles Tendinopathy,” Scandinavian Journal of Medicine & Science in Sports 29, no. 8 (2019): 1223–1231. 10.1111/sms.13450 31044450

[209] M. Dejnek , A. Królikowska , M. Kowal , et al., “Comparative Efficacy of Platelet‐Rich Plasma, Corticosteroid, Hyaluronic Acid, and Placebo (Saline) Injections in Patients With Lateral Elbow Tendinopathy: A Randomized Controlled Trial,” Journal of Clinical Medicine 14, no. 2 (2025): 472. 10.3390/jcm14020472 PMC11766060 39860479

[210] Y. He , S. Lu , W. Chen , et al., “Exosomes Derived From Tendon Stem/Progenitor Cells Enhance Tendon‐Bone Interface Healing After Rotator Cuff Repair in a Rat Model,” Bioactive Materials 40 (2024): 484–502. 10.1016/j.bioactmat.2024.06.014 PMC11260958 39040569

[211] D. Li , J. Jiu , H. Liu , et al., “Tissue‐engineered Mesenchymal Stem Cell Constructs Alleviate Tendinopathy by Suppressing Vascularization,” Bioactive Materials 36 (2024): 474–489. 10.1016/j.bioactmat.2024.06.029 PMC11269794 39055350

[212] T. Oshita , M. Tobita , S. Tajima , et al., “Adipose‐Derived Stem Cells Improve Collagenase‐Induced Tendinopathy in a Rat Model,” American Journal of Sports Medicine 44, no. 8 (2016): 1983–1989. 10.1177/0363546516640750 27159294

[213] S. Kokubu , R. Inaki , K. Hoshi , et al., “Adipose‐Derived Stem Cells Improve Tendon Repair and Prevent Ectopic Ossification in Tendinopathy by Inhibiting Inflammation and Inducing Neovascularization in the Early Stage of Tendon Healing,” Regenerative Therapy 14 (2020): 103–110. 10.1016/j.reth.2019.12.003 PMC6970144 31989000

[214] D. Deng , W. Wang , and B. Wang , “Repair of Achilles Tendon Defect With Autologous ASCs Engineered Tendon in a Rabbit Model,” Biomaterials 35, no. 31 (2014): 8801–8809. 10.1016/j.biomaterials.2014.06.058 25069604

[215] H. Pas , M. H. Moen , H. J. Haisma , et al., “No Evidence for the Use of Stem Cell Therapy for Tendon Disorders: A Systematic Review,” British Journal of Sports Medicine 51, no. 13 (2017): 996–1002. 10.1136/bjsports-2016-096794 28077355

[216] Z. Yuan , D. Yu , Y. Wang , et al., “Early Delivery of Human Umbilical Cord Mesenchymal Stem Cells Improves Healing in a Rat Model of Achilles Tendinopathy,” Regenerative Medicine 19, no. 2 (2024): 93–102. 10.2217/rme-2023-0222 38415316

[217] Z. Ge , W. Li , R. Zhao , et al., “Programmable DNA Hydrogel Provides Suitable Microenvironment for Enhancing TSPCS Therapy in Healing of Tendinopathy,” Small 19, no. 32 (2023): e2207231. 10.1002/smll.202207231 37066733

[218] Y. Dou , H. Zhai , H. Li , et al., “Endothelial Cells‐Derived Exosomes‐Based Hydrogel Improved Tendinous Repair via Anti‐Inflammatory and Tissue Regeneration‐promoting Properties,” Journal of Nanobiotechnology 22, no. 1 (2024): 401. 10.1186/s12951-024-02607-0 PMC11232200 38982446

[219] K. A. Alberti , C. I. Neufeld , J. Wang , et al., “In Vivo Peripheral Nerve Repair Using Tendon‐Derived Nerve Guidance Conduits,” ACS Biomaterials Science & Engineering 2, no. 6 (2016): 937–945. 10.1021/acsbiomaterials.6b00034 33429503

[220] G. Walden , X. Liao , S. Donell , et al., “A Clinical, Biological, and Biomaterials Perspective Into Tendon Injuries and Regeneration,” Tissue Engineering Part B: Reviews 23, no. 1 (2017): 44–58. 10.1089/ten.teb.2016.0181 PMC5312458 27596929

[221] W. Kim , G. E. Kim , M. Attia Abdou , et al., “Tendon‐Inspired Nanotopographic Scaffold for Tissue Regeneration in Rotator Cuff Injuries,” ACS Omega 5, no. 23 (2020): 13913–13925. 10.1021/acsomega.0c01328 PMC7301599 32566858

[222] G. C. Dosal , J. D. Schroeder , and R. C. Oh , “Low‐Volume Hydrodissection for the Treatment of Chronic Achilles Tendinopathy,” Military Medicine 188, no. 9‐10 (2023): e3269–e3272. 10.1093/milmed/usac384 36515159

[223] A. W. Ilaltdinov , Y. Gong , D. J. Leong , et al., “Advances in the Development of Gene Therapy, Noncoding RNA, and Exosome‐Based Treatments for Tendinopathy,” Annals of the New York Academy of Sciences 1490, no. 1 (2021): 3–12. 10.1111/nyas.14382 32501571

[224] A. V. September , J. Cook , C. J. Handley , et al., “Variants Within the COL5A1 Gene Are Associated With Achilles Tendinopathy in Two Populations,” British Journal of Sports Medicine 43, no. 5 (2009): 357–365. 10.1136/bjsm.2008.048793 18443036

[225] X. Wang , S. Liu , T. Yu , et al., “Inhibition of Integrin αvβ6 Activation of TGF‐β Attenuates Tendinopathy,” Advanced Science 9, no. 11 (2022): e2104469. 10.1002/advs.202104469 PMC9009110 35166070

[226] J. B. Tang , Y. F. Wu , Y. Cao , et al., “Basic FGF or VEGF Gene Therapy Corrects Insufficiency in the Intrinsic Healing Capacity of Tendons,” Scientific Reports 6 (2016): 20643. 10.1038/srep20643 PMC4749961 26865366

[227] D. Challoumas , P. D. Kirwan , D. Borysov , et al., “Topical Glyceryl Trinitrate for the Treatment of Tendinopathies: A Systematic Review,” British Journal of Sports Medicine 53, no. 4 (2019): 251–262. 10.1136/bjsports-2018-099552 PMC6362607 30301735

[228] Y. Assem and M. Arora , “Glyceryl Trinitrate Patches‐An Alternative Treatment for Shoulder Impingement Syndrome,” Journal of Orthopaedic Translation 3, no. 1 (2014): 12–20. 10.1016/j.jot.2014.11.001 PMC5982354 30035035

[229] L. Cheng , Q. Zheng , K. Qiu , et al., “Mitochondrial Destabilization in Tendinopathy and Potential Therapeutic Strategies,” Journal of Orthopaedic Translation 49 (2024): 49–61. 10.1016/j.jot.2024.09.003 PMC11488423 39430132

[230] S. P. Magnusson , H. Langberg , and M. Kjaer , “The Pathogenesis of Tendinopathy: Balancing the Response to Loading,” Nature Reviews Rheumatology 6, no. 5 (2010): 262–268. 10.1038/nrrheum.2010.43 20308995

[231] K. Li , J. Zhao , M. Wang , et al., “The Roles of Various Prostaglandins in Fibrosis: A Review,” Biomolecules 11, no. 6 (2021): 789. 10.3390/biom11060789 PMC8225152 34073892

[232] Z. Ma , A. Y. W. Lee , C. H. Kot , et al., “Upregulation of FABP4 Induced Inflammation in the Pathogenesis of Chronic Tendinopathy,” Journal of Orthopaedic Translation 47 (2024): 105–115. 10.1016/j.jot.2024.06.007 PMC11245957 39007036

[233] C. Tang , Z. Wang , Y. Xie , et al., “Classification of Distinct Tendinopathy Subtypes for Precision Therapeutics,” Nature Communications 15, no. 1 (2024): 9460. 10.1038/s41467-024-53826-w PMC11530571 39487125

[234] C. Mondini Trissino da Lodi , M. Salerno , G. Merli , et al., “Tendinopathy: Sex Bias Starts From the Preclinical Development of Tendon Treatments. A Systematic Review,” Biology of Sex Differences 13, no. 1 (2022): 44. 10.1186/s13293-022-00453-z PMC9338527 35908065

[235] N. L. Millar , I. B. McInnes , F. Kolbinger , et al., “Targeting the IL‐17A Pathway for Therapy in Early‐Stage Tendinopathy,” RMD Open 11, no. 1 (2025): e004729. 10.1136/rmdopen-2024-004729 PMC11881027 39988349

[236] C. Kudron , M. J. Carlson , A. Meron , et al., “Using Ultrasound Measurement of the Achilles Tendon in Asymptomatic Runners to Assist in Predicting Tendinopathy,” Journal of Ultrasound in Medicine 39, no. 3 (2020): 491–496. 10.1002/jum.15125 31490583

[237] K. Holt , A. Delbridge , L. Josey , et al., “Subscapularis Tendinopathy Is Highly Prevalent in Elite Swimmer's Shoulders: An MRI Study,” Journal of Science and Medicine in Sport 25, no. 9 (2022): 720–725. 10.1016/j.jsams.2022.06.010 35906124

[238] Y. X. Zhu , J. Q. Huang , Y. Y. Ming , et al., “Screening of Key Biomarkers of Tendinopathy Based on Bioinformatics and Machine Learning Algorithms,” PLoS ONE 16, no. 10 (2021): e0259475. 10.1371/journal.pone.0259475 PMC8555777 34714891

[239] M. E. Dancy , A. S. Alexander , C. J. Clark , et al., “Gluteal Tendinopathy: Critical Analysis Review of Current Nonoperative Treatments,” JBJS Reviews 11, no. 10 (2023): e23.00101. 10.2106/JBJS.RVW.23.00101 37812677

[240] D. Metcalfe , J. Achten , and M. L. Costa , “Glucocorticoid Injections in Lesions of the Achilles Tendon,” Foot & Ankle International 30, no. 7 (2009): 661–665. 10.3113/FAI.2009.0661 19589313

[241] D. Kouroupis , C. Perucca Orfei , D. Correa , et al., “Cellular and Structural Changes in Achilles and Patellar Tendinopathies: A Pilot in Vivo Study,” Biomedicines 12, no. 5 (2024): 995. 10.3390/biomedicines12050995 PMC11117798 38790957

[242] K. Kulig , Y. J. Chang , and D. A. Ortiz‐Weissberg , “Perspective on Reversibility of Tendinosis‐Induced Multi‐Level Adaptations,” Frontiers in Physiology 11 (2020): 651. 10.3389/fphys.2020.00651 PMC7358446 32733262

[243] Y. Zhang , X. Li , H. Li , et al., “Anti‐inflammatory Therapy for Tendinopathy Using Il1rn mRNA Encapsulated in SM102 Lipid Nanoparticles,” Frontiers in Bioengineering and Biotechnology 13 (2025): 1641236. 10.3389/fbioe.2025.1641236 PMC12378938 40873435

[244] M. G. Raimondo , H. Mohammadian , S. Rauber , et al., “Entheseal Tissue Signature in Response to IL‐17A Inhibition in Psoriatic Arthritis: Results From the EBIO Entheseal Biopsy Study,” Annals of the Rheumatic Diseases 85, no. 2 (2026): 285–296. 10.1016/j.ard.2025.08.032 41107119

[245] V. Arvind , G. Crosio , K. Howell , et al., “Functional Tendon Regeneration Is Driven by Regulatory T Cells and IL‐33 Signaling,” Science Advances 11, no. 17 (2025): eadn5409. 10.1126/sciadv.adn5409 PMC12017337 40267206

[246] Y. Yu , Y. Zhou , T. Cheng , et al., “Hypoxia Enhances Tenocyte Differentiation of Adipose‐Derived Mesenchymal Stem Cells by Inducing Hypoxia‐Inducible Factor‐1α in a Co‐Culture System,” Cell Proliferation 49, no. 2 (2016): 173–184. 10.1111/cpr.12250 PMC6496829 27021233

[247] S. Agarwal , S. Loder , C. Brownley , et al., “Inhibition of Hif1α Prevents both Trauma‐Induced and Genetic Heterotopic Ossification,” PNAS 113, no. 3 (2016): E338–E347. 10.1073/pnas.1515397113 PMC4725488 26721400

[248] R. C. Poulsen , H. J. Knowles , A. J. Carr , et al., “Cell Differentiation versus Cell Death: Extracellular Glucose Is a Key Determinant of Cell Fate Following Oxidative Stress Exposure,” Cell Death & Disease 5, no. 2 (2014): e1074. 10.1038/cddis.2014.52 PMC3944267 24556689

[249] L. Lei , Z. Wen , M. Cao , et al., “The Emerging Role of Piezo1 in the Musculoskeletal System and Disease,” Theranostics 14, no. 10 (2024): 3963–3983. 10.7150/thno.96959 PMC11234281 38994033

[250] R. Nakamichi , S. Ma , T. Nonoyama , et al., “The Mechanosensitive Ion Channel PIEZO1 Is Expressed in Tendons and Regulates Physical Performance,” Science Translational Medicine 14, no. 647 (2022): eabj5557. 10.1126/scitranslmed.abj5557 35648809

[251] W. Hu , J. Yang , and L. Liu , Exploring Trends and Gaps in Osteoarthritis Biomarker Research (1999‐2024) (A Citation Analysis of Top 50 Cited Articles), 10.1177/19476035241288660. PMC11556567 39422972

[252] W. Morita , S. G. Dakin , S. J. B. Snelling , et al., “Cytokines in Tendon Disease: A Systematic Review,” Bone & Joint Research 6, no. 12 (2017): 656–664. 10.1302/2046-3758.612.BJR-2017-0112.R1 PMC5935810 29203638

[253] R. Chen , L. Ai , J. Zhang , et al., “Dendritic Cell‐Derived Exosomes Promote Tendon Healing and Regulate Macrophage Polarization in Preventing Tendinopathy,” International Journal of Nanomedicine 19 (2024): 11701–11718. 10.2147/IJN.S466363 PMC11571930 39558915

[254] X. Cheng , X. Yun , Y. Wei , et al., “Hypoxia‐Mimicking Microenvironment Scaffold for Enhanced Tendon Regeneration,” ACS Applied Materials & Interfaces 17, no. 6 (2025): 8937–8948. 10.1021/acsami.4c18082 39901352

[255] G. Nourissat , F. Berenbaum , and D. Duprez , “Tendon Injury: From Biology to Tendon Repair,” Nature Reviews Rheumatology 11, no. 4 (2015): 223–233. 10.1038/nrrheum.2015.26 25734975

[256] S. H. Han , H. K. Kim , Y. Jang , et al., “The Expression of Substance P and Calcitonin Gene‐related Peptide Is Associated With the Severity of Tendon Degeneration in Lateral Epicondylitis,” BMC Musculoskeletal Disorders 22, no. 1 (2021): 210. 10.1186/s12891-021-04067-1 PMC7898744 33612098

[257] J. Zhang and J. H. Wang , “The Effects of Mechanical Loading on Tendons–an in Vivo and in Vitro Model Study,” PLoS ONE 8, no. 8 (2013): e71740. 10.1371/journal.pone.0071740 PMC3747237 23977130

[258] Y. T. Wu , Y. T. Wu , T. C. Huang , et al., “Sequential Inflammation Model for Achilles Tendinopathy by Elastin Degradation With Treadmill Exercise,” Journal of Orthopaedic Translation 23 (2020): 113–121. 10.1016/j.jot.2020.03.004 PMC7322491 32642426

[259] M. Pierantoni , K. Sharma , J. Kok , et al., “Quantification of 3D Microstructures in Achilles Tendons During in Situ Loading Reveals Anisotropic fiber Response,” Acta Biomaterialia 194 (2025): 246–257. 10.1016/j.actbio.2025.01.023 39800097

[260] J. Luo , Z. Wang , C. Tang , et al., “Animal Model for Tendinopathy,” Journal of Orthopaedic Translation 42 (2023): 43–56. 10.1016/j.jot.2023.06.005 PMC10450357 37637777

[261] G. Rodas , L. Osaba , D. Arteta , et al., “Genomic Prediction of Tendinopathy Risk in Elite Team Sports,” International Journal of Sports Physiology and Performance 15, no. 4 (2019): 489–495. 10.1123/ijspp.2019-0431 31615970

[262] S. S. Desai , J. D. Mueller , T. T. Wong , et al., “Preseason Patellar Tendon Thickness Predicts Symptomatic Patellar Tendinopathy in Male NCAA Division I Basketball Players,” Journal of Bone and Joint Surgery American Volume 107, no. 10 (2025): e51. 10.2106/JBJS.24.00680 40100945

[263] F. Sarto , J. Spörri , D. P. Fitze , et al., “Implementing Ultrasound Imaging for the Assessment of Muscle and Tendon Properties in Elite Sports: Practical Aspects, Methodological Considerations and Future Directions,” Sports Medicine 51, no. 6 (2021): 1151–1170. 10.1007/s40279-021-01436-7 PMC8124062 33683628

[264] S. S. Rudisill , N. H. Varady , M. P. Kucharik , et al., “Evidence‐Based Hamstring Injury Prevention and Risk Factor Management: A Systematic Review and Meta‐analysis of Randomized Controlled Trials,” American Journal of Sports Medicine 51, no. 7 (2023): 1927–1942. 10.1177/03635465221083998 35384731

[265] F. Mersmann , S. Bohm , T. Domroes , et al., “Personalized Injury Risk Assessment and Exercise Prescription Based on Tendon Strain,” Exercise and Sport Sciences Reviews 53, no. 2 (2025): 77–86. 10.1249/JES.0000000000000359 39919310

[266] A. C. van der Vlist , S. J. Breda , E. H. G. Oei , et al., “Clinical Risk Factors for Achilles Tendinopathy: A Systematic Review,” British Journal of Sports Medicine 53, no. 21 (2019): 1352–1361. 10.1136/bjsports-2018-099991 PMC6837257 30718234

[267] S. J. Kim , P. D. Tatman , D. H. Song , et al., “Nanotopographic Cues and Stiffness Control of Tendon‐Derived Stem Cells From Diverse Conditions,” International Journal of Nanomedicine 13 (2018): 7217–7227. 10.2147/IJN.S181743 PMC6231514 30510414

[268] P. P. Lui and S. W. Ng , “Cell Therapy for the Treatment of Tendinopathy–a Systematic Review on the Pre‐Clinical and Clinical Evidence,” Seminars in Arthritis and Rheumatism 42, no. 6 (2013): 651–666. 10.1016/j.semarthrit.2012.10.004 23369658

[269] D. Ruan , Y. Fei , S. Qian , et al., “Early‐Stage Primary Anti‐inflammatory Therapy Enhances the Regenerative Efficacy of Platelet‐Rich Plasma in a Rabbit Achilles Tendinopathy Model,” American Journal of Sports Medicine 49, no. 12 (2021): 3357–3371. 10.1177/03635465211037354 34549620

[270] B. Vicenzino , R. J. de Vos , H. Alfredson , et al., “ICON 2019‐International Scientific Tendinopathy Symposium Consensus: There Are Nine Core Health‐Related Domains for Tendinopathy (CORE DOMAINS): Delphi Study of Healthcare Professionals and Patients,” British Journal of Sports Medicine 54, no. 8 (2020): 444–451. 10.1136/bjsports-2019-100894 31685525

[271] A. S. Agergaard , R. B. Svensson , N. M. Malmgaard‐Clausen , et al., “Clinical Outcomes, Structure, and Function Improve with both Heavy and Moderate Loads in the Treatment of Patellar Tendinopathy: A Randomized Clinical Trial,” American Journal of Sports Medicine 49, no. 4 (2021): 982–993. 10.1177/0363546520988741 33616456

[272] S. W. Jhan , K. T. Wu , W. Y. Chou , et al., “A Comparative Analysis of Platelet‐Rich Plasma Alone versus Combined With Extracorporeal Shockwave Therapy in Athletes With Patellar Tendinopathy and Knee Pain: A Randomized Controlled Trial,” Knee Surgery & Related Research 36, no. 1 (2024): 47. 10.1186/s43019-024-00252-3 PMC11650825 39690426

[273] S. Moosmayer , G. Lund , U. S. Seljom , et al., “Fifteen‐Year Results of a Comparative Analysis of Tendon Repair versus Physiotherapy for Small‐to‐Medium‐Sized Rotator Cuff Tears: A Concise Follow‐Up of Previous Reports,” Journal of Bone and Joint Surgery American Volume 106, no. 19 (2024): 1785–1796. 10.2106/JBJS.24.00065 PMC11594065 39197154

[274] B. H. Kjær , S. P. Magnusson , M. Henriksen , et al., “Effects of 12 Weeks of Progressive Early Active Exercise Therapy after Surgical Rotator Cuff Repair: 12 Weeks and 1‐Year Results from the CUT‐N‐MOVE Randomized Controlled Trial,” American Journal of Sports Medicine 49, no. 2 (2021): 321–331. 10.1177/0363546520983823 33471547

[275] S. E. Harper , R. A. Roembke , J. D. Zunker , et al., “Wearable Tendon Kinetics,” Sensors (Basel) 20, no. 17 (2020): 4805. 10.3390/s20174805 PMC7506797 32858833

[276] R. Prado‐Costa , J. Rebelo , J. Monteiro‐Barroso , et al., “Ultrasound Elastography: Compression Elastography and Shear‐wave Elastography in the Assessment of Tendon Injury,” Insights Imaging 9, no. 5 (2018): 791–814. 10.1007/s13244-018-0642-1 PMC6206379 30120723

[277] J. R. Dahlqvist , P. Widholm , O. D. Leinhard , et al., “MRI in Neuromuscular Diseases: An Emerging Diagnostic Tool and Biomarker for Prognosis and Efficacy,” Annals of Neurology 88, no. 4 (2020): 669–681. 10.1002/ana.25804 32495452

[278] M. Huang , H. Yu , R. Gao , et al., “Photoacoustic Imaging in Inflammatory Orthopedic Diseases: Progress Toward Precise Diagnostics and Predictive Regulation,” Advanced Science 12, no. 14 (2025): e2412745. 10.1002/advs.202412745 PMC11984849 40019846

[279] Y. Noda , K. Sekiguchi , S. Matoba , et al., “Real‐Time Artificial Intelligence‐Based Texture Analysis of Muscle Ultrasound Data for Neuromuscular Disorder Assessment,” Clinical Neurophysiology Practice 9 (2024): 242–248. 10.1016/j.cnp.2024.08.003 PMC11402302 39282049

[280] Y. Meng , M. Wang , N. Niu , et al., “Artificial Intelligence‐Assisted Diagnosis of Early Allograft Dysfunction Based on Ultrasound Image and Data,” Visual Computing for Industry, Biomedicine, and Art 8, no. 1 (2025): 13. 10.1186/s42492-025-00192-z PMC12069173 40353942

[281] J. Chang , J. Li , J. Ye , et al., “AI‐Enabled Piezoelectric Wearable for Joint Torque Monitoring,” Nano‐Micro Letters 17, no. 1 (2025): 247. 10.1007/s40820-025-01753-w PMC12048387 40316813

[282] M. P. Kwon , T. J. Hullfish , C. J. Humbyrd , et al., “Wearable Sensor and Machine Learning Estimate Tendon Load and Walking Speed During Immobilizing Boot Ambulation,” Scientific Reports 13, no. 1 (2023): 18086. 10.1038/s41598-023-45375-x PMC10593749 37872320

[283] S. T. Oskouei , P. Malliaras , K. D. Hill , et al., “Monitoring Physical Activity Using Wearable Technology in People With Achilles Tendinopathy Undergoing Physiotherapy Treatment: A Feasibility Prospective Cohort Study,” Physiotherapy 120 (2023): 38–46. 10.1016/j.physio.2023.04.001 37364446

[284] Y. C. Lai , Y. C. Kan , Y. C. Lin , et al., “AIoT‐Enabled Rehabilitation Recognition System‐Exemplified by Hybrid Lower‐Limb Exercises,” Sensors (Basel) 21, no. 14 (2021): 4761. 10.3390/s21144761 PMC8309886 34300501

[285] R. De Fazio , V. M. Mastronardi , M. De Vittorio , et al., “Wearable Sensors and Smart Devices to Monitor Rehabilitation Parameters and Sports Performance: An Overview,” Sensors (Basel) 23, no. 4 (2023): 1856. 10.3390/s23041856 PMC9965388 36850453

[286] K. Xiang , M. Liu , J. Chen , et al., “AI‐Assisted Insole Sensing System for Multifunctional Plantar‐Healthcare Applications,” ACS Appl Mater Interfaces 16, no. 25 (2024): 32662–32678. 10.1021/acsami.4c04467 38863342

[287] D. S. Morais , J. Torres , R. M. Guedes , et al., “Current Approaches and Future Trends to Promote Tendon Repair,” Annals of Biomedical Engineering 43, no. 9 (2015): 2025–2035. 10.1007/s10439-015-1369-5 26122512

[288] C. Shen and A. Shen , “D Printing: Innovative Solutions and Technological Advances in Orthopedic Repair and Reconstruction, Personalized Treatment and Drug Delivery,” Biomedical Engineering Online 24, no. 1 (2025): 5. 10.1186/s12938-025-01334-3 PMC11748259 39838448

[289] A. Tebani , C. Afonso , S. Marret , et al., “Omics‐Based Strategies in Precision Medicine: Toward a Paradigm Shift in Inborn Errors of Metabolism Investigations,” International Journal of Molecular Sciences 17, no. 9 (2016): 1555. 10.3390/ijms17091555 PMC5037827 27649151

[290] S. K. Niazi , “Molecular Biosimilarity‐An AI‐Driven Paradigm Shift,” International Journal of Molecular Sciences 23, no. 18 (2022): 10690. 10.3390/ijms231810690 PMC9505197 36142600

